# Temporal Trend and Health Inequality in the Burden of Autoimmune Diseases Among Older Adolescents and Young Adults Aged 15-29 Years

**DOI:** 10.5152/ArchRheumatol.2025.11174

**Published:** 2025-09-01

**Authors:** Sheng Li, Yan-yu Zhu, Harry Asena Musonye, Qian-qian Shi, Hai-fen Wei, Shu-shan Zhao, Hai-feng Pan, Peng Wang

**Affiliations:** 1 Department of Health Promotion and Behavioral Sciences, Anhui Medical University School of Public Health, Hefei, China; 2 Department of Epidemiology and Biostatistics, Anhui Medical University School of Public Health, Hefei, China; 3 Department of Rheumatology and Immunology, Shaoxing People’s Hospital, Zhejiang, China

**Keywords:** Autoimmune diseases, disease burden, older adolescents, young adults

## Abstract

**Background/Aims:**

Autoimmune diseases (ADs) are a group of disorders characterized by the dysfunction of the immune system, leading to self-directed attacks on organs or tissues. The global burden of ADs in older adolescents and young adults is still lacking and requires updates.This study described the global, regional, and country-specific disease burden and temporal trends of ADs in older adolescents and young adults (aged 15-29 years) from 1990 to 2019.

**Materials and Methods:**

Data from the 2019 Global Burden of Disease, Injury, and Risk Factors study were utilized to report age-standardized incidence rate (ASIR), age-standardized prevalence rate (ASPR), age-standardized mortality rate (ASMR), and age-standardized disability-adjusted life years (ASDR) rates of ADs at global, regional, and national levels. The average annual percent change was determined by Joinpoint regression analysis.

**Results:**

In 2019, the burden of alopecia areata (AA) and rheumatic heart disease (RHD) in older adolescents and young adults was particularly notable. Specifically, the ASIR for AA was 493.84 per 100 000 (95% uncertainty interval (UI): 444.13, 544.49), while the ASPR, ASMR, and ASDR for RHD were 771.43 per 100 000 (95% UI: 529.38, 1074.04), 1.08 per 100 000 (95% UI: 0.94, 1.23), and 108.36 per 100 000 (95% UI: 88.63, 133.67), respectively. From 1990 to 2019, the heavy disease burden of ADs was more pronounced in the European region and region of the Americas, where Italy and El Salvador were particularly affected. Although the burden of ADs was generally more severe in females than in males across most regions, males consistently had higher ASIR (10.19 per 100 000; 95% UI: 4.68, 18.22), ASPR (249.77 per 100 000; 95% UI: 186.89, 325.98), ASMR (0.53 per 100 000; 95% UI: 0.47, 0.60), and ASDR (47.59 per 100 000; 95% UI: 40.97, 56.34) for type 1 diabetes mellitus (T1DM) compared to females.

**Conclusion:**

Globally, there is an increasing burden of AA, T1DM, and RHD in older adolescents and young adults. The American and European regions and females endure a severe burden of ADs. Healthcare providers should be aware of the heavy burden of ADs and develop age-appropriate prevention, diagnosis, and intervention strategies to achieve health equity.

**Main Points**

- Rheumatic heart disease showed the highest global burden among autoimmune diseases in 2019, while alopecia areata had the highest incidence.
- From 1990 to 2019, the incidence and prevalence of type 1 diabetes mellitus,rheumatoid arthritis, and rheumatic heart disease increased globally.
- Females bore higher burdens for most autoimmune diseases, whereas type 1 diabetes mellitus was more prevalent and fatal in males.
- Regional disparities were marked, with the highest burden observed in Europe and the Americas, and the lowest in Southeast Asia.

## Introduction

Autoimmune diseases (ADs) are a diverse group of systemic immune-mediated disorders characterized by aberrant immune response against self-antigens, leading to chronic inflammation and subsequent tissue or organ damage.^1^ Globally, ADs affect approximately 3%-5% of the population with a notable sex disparity, where females are disproportionately affected, exhibiting a female-to-male ratio as high as 10 : 1.^2^ The rising global burden of ADs impacts not only physical and psychological well-being but also imposes substantial and escalating challenges on healthcare systems, families, and communities.^3^

Although ADs can manifest at any age, growing evidence suggests an increasing incidence among children, adolescents, and young adults.^4^ Of particular concern is the fact that ADs have become a leading cause of chronic morbidity and even mortality among young and middle-aged women.^5^ Conditions such as inflammatory bowel disease (IBD) and juvenile idiopathic arthritis impose significant long-term health burdens on children and adolescents.^6,7^ This trend is especially alarming in low- and middle-income countries, where the rapidly growing youth population further accentuates the need for targeted health interventions.^8^

Older adolescents and young adults represent a transitional life stage marked by significant physiological, psychological, and social changes. This period is often associated with delayed diagnosis, suboptimal disease management, and unique healthcare challenges.^9,10^ However, global epidemiological studies on ADs have predominantly focused on adults or the elderly, resulting in limited data and underrepresentation of this crucial age group.^11^ The underrepresentation of this demographic in AD surveillance may hinder the timely implementation of effective prevention, diagnosis, and treatment strategies. Therefore, understanding the epidemiological patterns and disease burden of ADs in this age group is essential to inform age-specific interventions and policy-making.

The Global Burden of Disease, Injuries, and Risk Factors (GBD) 2019 study offers a robust and comprehensive platform for evaluating temporal trends and regional patterns of disease burden across countries and population.^12^ By incorporating standardized methods and time-based health metrics, GBD 2019 enables comparative epidemiological analyses and supports evidence-based health policy development.^13,14^

In the present study, data from GBD 2019 were used to systematically evaluate the global, regional, and national burdens of 7 major ADs, including rheumatoid arthritis (RA), IBD, multiple sclerosis (MS), type 1 diabetes mellitus (T1DM), rheumatic heart disease (RHD), psoriasis, and alopecia areata (AA), in older adolescents and young adults (aged 15 to 29 years) from 1990 to 2019. By identifying the temporal patterns and high-burden regions, this study can provide a population-based foundation for developing targeted public health policies, enhancing early recognition, and supporting resource allocation for this underserved population group.

## Methods

### Data Sources and Definitions

This study utilized data from GBD 2019, coordinated by the Institute for Health Metrics and Evaluation. The GBD 2019 provides systematic estimates for 369 diseases and injuries, 87 risk factors, and 204 countries and territories from 1990 to 2019. The data were derived from a wide range of sources, including vital registration systems, population-based surveys, disease registries, hospital records, and published literature. Mortality estimates were generated using the Cause of Death Ensemble model, while non-fatal health outcomes such as incidence, prevalence, and years lived with disability (YLDs) were estimated using DisMod-MR 2.1, a Bayesian meta-regression modeling tool.^15 ^

The key burden metrics included incidence, prevalence, mortality, years of life lost (YLLs), YLDs, and disability-adjusted life years (DALYs). All metrics were age-standardized using the GBD global standard population and reported as per 100 000 population to ensure comparability across regions and time.^16^ To quantify uncertainty, it uses 1000 draw-level simulations for each estimate, and 95% uncertainty intervals (UIs) were calculated as the 2.5th and 97.5th percentiles of the posterior distribution.

The DALYs were calculated as the sum of YLLs due to premature mortality and YLDs due to disability. In addition, the age-standardized incidence rate (ASIR), age-standardized prevalence rate (ASPR), age-standardized mortality rate (ASMR), and age-standardized disability-adjusted life years (ASDR) for each disease across countries, sexes, and years were calculated according to the previous reports of standard GBD protocols.^15,17^

### Disease Classification and Diagnosis

Seven ADs were included in the analysis based on relevance, data completeness, and standardized definitions available within the GBD framework. The disease classification relied on the International Classification of Diseases, 10th revision codes: RA (M05-M05.9 or M08-M09.8), IBD (K50-K51.319, K51.5-K52, and K52.8-K52.9), MS (G35-G35.0), T1DM (E10-E10.11 and E10.3-E10.9), RHD (I01-I01.9, I02.0, and I05-I09.9), psoriasis (L40-L41.9), and AA (L63-L63.9). These diseases were identified based on the cause list mapping of GBD, which ensures consistency in disease definitions across regions and health systems, despite potential variability in diagnostic capacity and healthcare access.^18^

### Study Population and Age Stratification

The study focused on older adolescents and young adults, following the GBD standard age groups. The older adolescents were categorized as individuals aged 15-19 years, and young adults were defined as aged 20-29 years. Specifically, to maintain a similar period with the older adolescents group, young adults were then stratified into 2 subgroups: 20-24 years and 25-29 years.^19^ This categorization aligns with established demographic frameworks and facilitates comparative analyses across different age groups and sexes.

### Statistical Analysis

Data on ASIR, ASPR, ASMR, and ASDR for each ADs were extracted from the GBD 2019 study across 204 countries and territories from 1990 to 2019. A direct age-standardized method was applied, assuming that the input metrics follow a Poisson distribution.^20,21^

Trend analysis was performed using Joinpoint regression modeling to identify statistically significant changes over time.^22^ Temporal trends were examined over 3 intervals (1990-1999, 2000-2009, and 2010-2019) by calculating the average annual percent change (AAPC) and 95% UIs. The model parameters included: minimum number of joinpoints: 0; maximum number of joinpoints: 3; model selection method: Bayesian information criterion. An AAPC with a 95% UI entirely above 0 was interpreted as a statistically significant increasing trend, while an AAPC with a UI entirely below 0 indicated a significant decreasing trend.

All statistical analyses and data visualizations were conducted using R software (version 4.3.2) (R Foundation for Statistical Computing; Vienna, Austria) and Joinpoint regression program software Version 5.3.0.0 (National Cancer Institute; Bethesda, MD, USA) 4.9.1.0 (National Cancer Institute; Bethesda, MD, USA). Given the use of anonymous, publicly available open data, no ethical approval or informed consent was required for this study. The 2-sided *P* < .05 was denoted for statistical significance.

## Results

### Global Trends of Autoimmune Diseases from 1990 to 2019

In 2019, AA exhibited the highest global ASIR at 493.84 per 100 000 population (95% UI: 444.13, 544.49). In contrast, RHD had the highest burden overall, with an ASPR of 771.43 per 100 000 (95% UI: 529.38, 1074.04), ASMR of 1.08 per 100 000 (95% UI: 0.94, 1.23), and ASDR of 108.36 per 100 000 (95% UI: 88.63, 133.67) (Table 1).

Sex-specific differences were observed across all metrics. Females had higher ASIR and ASPR than males for AA, MS, psoriasis, RHD, and RA. In contrast, T1DM showed a higher ASIR in males. Although the ASPR of IBD was higher in females, the ASIR remained higher in males. Regarding mortality and disability, females had a higher ASMR for IBD and MS, but a lower ASMR for T1DM and RHD. The ASDR was generally higher in females for most ADs, with the exception of T1DM (Table 1).

From 1990 to 2019, global ASIR and ASPR increased for T1DM, RHD, and RA. In contrast, AA, IBD, and psoriasis demonstrated declining trends in incidence and prevalence (Table 2). MS presented a complex pattern: although its global ASPR slightly declined, the ASIR increased in females, and its ASMR decreased across both sexes (Supplementary Figures 1-3). The ASDR for AA, IBD, MS, psoriasis, and RHD showed gradual global declines (Supplementary Figure 4). However, the AAPC of T1DM ASMR in females increased, while it decreased in males. Conversely, the ASDR of T1DM decreased in females but rose slightly in males (Supplementary Tables 1).

### Regional Trends of Autoimmune Diseases from 1990 to 2019

In 2019, the highest regional ASIR and ASPR were observed in Europe and the Americas, while the lowest were observed in Southeast Asia (Table 1). Although regional ASMR was relatively stable, ASDR exhibited substantial variability, especially for RHD. In the Eastern Mediterranean, RHD had an ASMR of 2.11 per 100 000 (95% UI: 1.55, 2.77) and an ASDR of 178.44 per 100 000 (95% UI: 136.07, 226.71); similar high burdens were observed in South-East Asia (ASMR: 2.09 per 100 000, 95% UI: 1.76, 2.47; ASDR: 175.27 per 100 000, 95% UI: 146.30, 209.34).

Females consistently had higher burdens for AA, MS, psoriasis, and RA across most regions (Table 1). The T1DM consistently imposed a greater burden in males. For IBD, regional sex disparities were noted: in the Eastern Mediterranean, females had higher ASPR (14.67 per 100 000, 95% UI: 10.9, 19.24) and ASMR (0.08 per 100 000, 95%UI: 0.04, 0.14); in the Americas, the similar higher burdens were found in females, with ASPR of 38.4 per 100,000 (95%UI: 32.52, 44.84) and ASMR of 0.07 per 100,000 (95%UI: 0.06, 0.08), respectively (Table 1).

From 1990 to 2019, T1DM burden increased in all regions, especially in the Western Pacific and Southeast Asia between 2000 and 2009. In contrast, IBD and MS showed declining trends in the Americas. Psoriasis showed global declines, but the European region exhibited a U-shaped trend in ASDR of psoriasis. The RA and RHD burdens showed significant decreases in Africa and the Western Pacific, respectively (Table 2). Notably, females showed more dynamic changes in RA and psoriasis, while males had greater variation in T1DM and IBD (Supplementary Tables 2).

### Country-Level Trends of Autoimmune Diseases from 1990 to 2019

**Figure 1.**
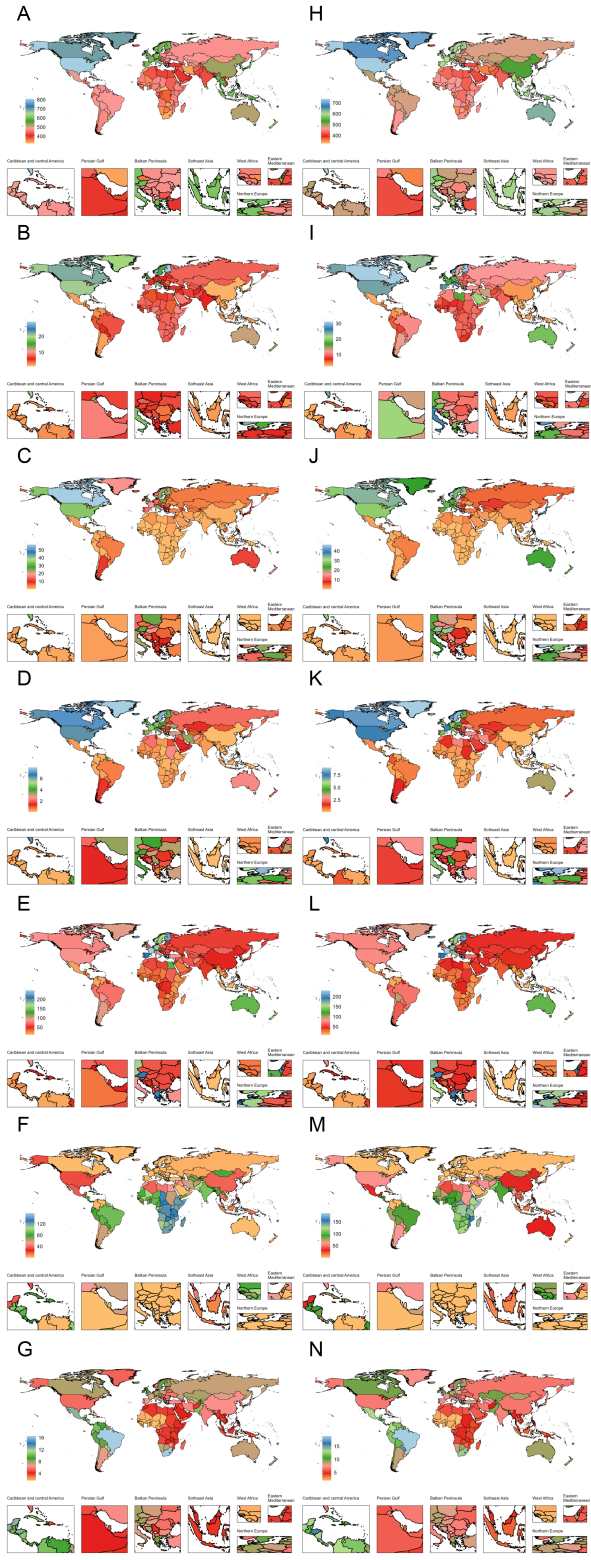
National age-standardized incidence rates (per 100 000) of 7 ADs among older adolescents and young adults in 1990 and 2019. ADs, autoimmune diseases; A, alopecia areata (AA); B, type 1 diabetes mellitus (T1DM); C, inflammatory bowel disease (IBD); D, multiple sclerosis (MS); E, psoriasis; F, rheumatic heart disease (RHD); G, rheumatoid arthritis (RA); H, alopecia areata (AA); I, type 1 diabetes mellitus (T1DM); J, inflammatory bowel disease (IBD); K, multiple sclerosis (MS); L, psoriasis; M, rheumatic heart disease (RHD); N, rheumatoid arthritis (RA); Panels A-G represent data for 1990; Panels H-N represent data for 2019.

**Figure 2.**
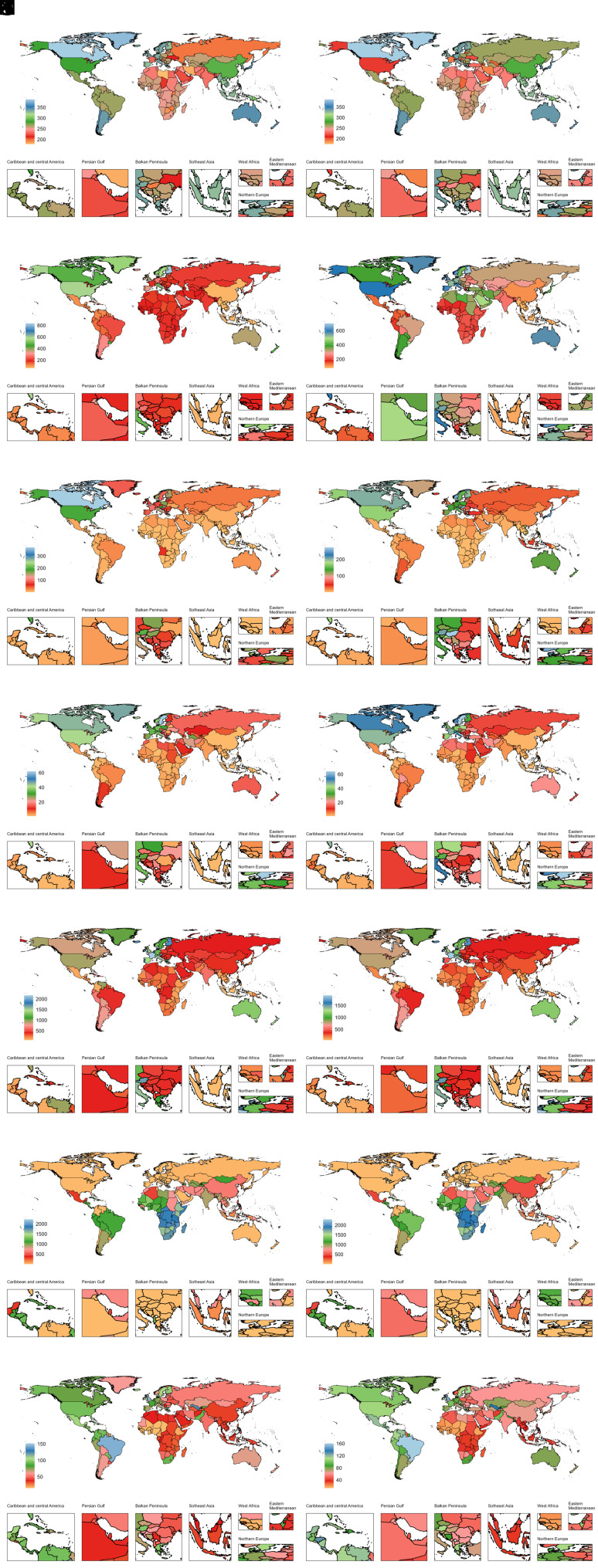
National age-standardized prevalence rates (per 100 000) of 7 ADs among older adolescents and young adults in 1990 and 2019. ADs, autoimmune diseases; A, alopecia areata (AA); B, type 1 diabetes mellitus (T1DM); C, inflammatory bowel disease (IBD); D, multiple sclerosis (MS); E, psoriasis; F, rheumatic heart disease (RHD); G, rheumatoid arthritis (RA); H, alopecia areata (AA); I, type 1 diabetes mellitus (T1DM); J, inflammatory bowel disease (IBD); K, multiple sclerosis (MS); L, psoriasis; M, rheumatic heart disease (RHD); N, rheumatoid arthritis (RA); Panels A-G represent data for 1990; Panels H-N represent data for 2019.

**Figure 3.**
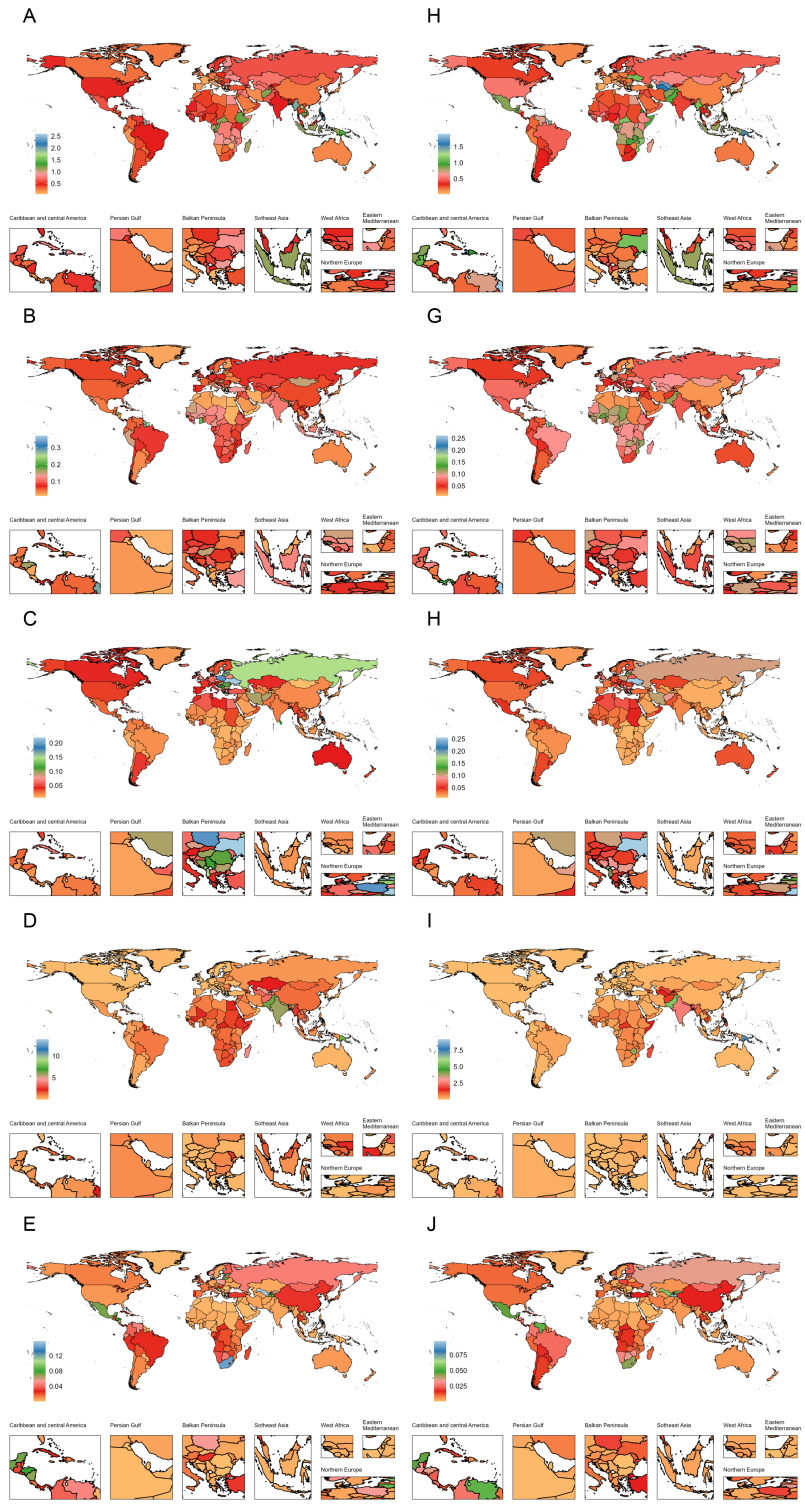
National age-standardized mortality rates (per 100 000) of 5 ADs among older adolescents and young adults in 1990 and 2019. ADs, autoimmune diseases; A, type 1 diabetes mellitus (T1DM); B, inflammatory bowel disease (IBD); C, multiple sclerosis (MS); D, rheumatic heart disease (RHD); E, rheumatoid arthritis (RA); F, type 1 diabetes mellitus (T1DM); G, inflammatory bowel disease (IBD); H, multiple sclerosis (MS); I, rheumatic heart disease (RHD); J, rheumatoid arthritis (RA); Panels A-E: 1990; Panels F-J: 2019.

**Figure 4.**
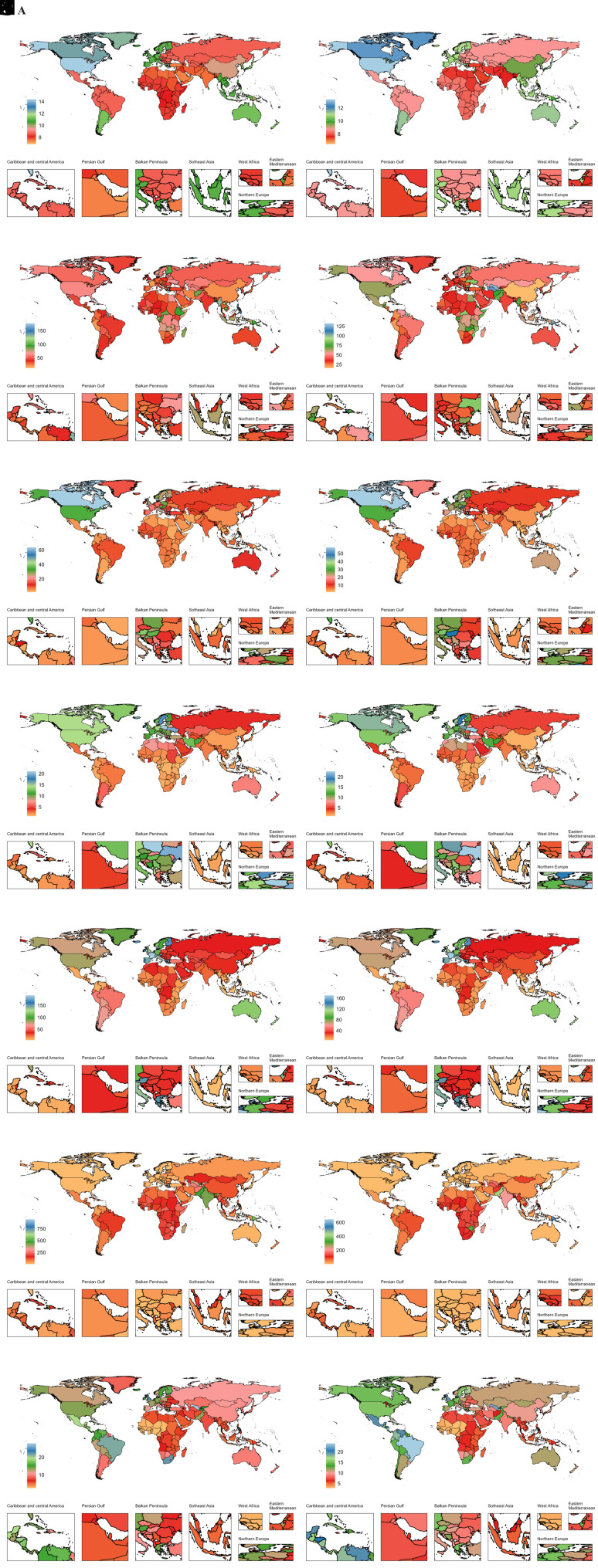
National age-standardized DALY rates (per 100 000) for 7 ADs among older adolescents and young adults in 1990 and 2019. ADs, autoimmune diseases; DALYs, disability-adjusted life years; A, alopecia areata (AA); B, type 1 diabetes mellitus (T1DM); C, inflammatory bowel disease (IBD); D, multiple sclerosis (MS); E, psoriasis; F, rheumatic heart disease (RHD); G, rheumatoid arthritis (RA); H, alopecia areata (AA); I, type 1 diabetes mellitus (T1DM); J, inflammatory bowel disease (IBD); K, multiple sclerosis (MS); L, psoriasis; M, rheumatic heart disease (RHD); N, rheumatoid arthritis (RA); Panels A-G: 1990; Panels H-N: 2019.

In 2019, the highest country-specific disease burdens of AA, T1DM, IBD, MS, psoriasis, RHD, and RA were observed in the United States, Finland, the Kingdom of Norway, Sweden, France, Somalia, and Brazil, respectively (Figures 1-4). Females generally had higher burdens for all ADs, except for T1DM and IBD, where male burdens were often comparable or higher (Supplementary Tables 3-6). Notably, El Salvador recorded the highest ASDR for T1DM among females at 165.66 per 100 000 (95% UI: 98.02, 256.48), and Cambodia reported the highest female ASDR for IBD at 37.16 per 100 000 (95% UI: 23.95, 53.76).

Although global trends remained stable, some countries exhibited significant shifts. Italy, El Salvador, and the United States showed sharper temporal changes in ADs burden (Supplementary Tables S7). At the country level, females consistently experienced a higher disease burden, although certain male-specific increases were observed. For example, males in Bhutan, Czechia, and Togo experienced increasing AA burden (Supplementary Figures 5-8). The ASPR of T1DM increased among Argentine females, and the ASDR of IBD was higher in Mauritian females (Supplementary Figures 9-10). For RA, male-specific increasing trends in ASIR, ASPR, and ASDR were observed in El Salvador, the Dominican Republic, and Serbia (Supplementary Figures 11-12).

## Discussion

This study comprehensively assessed the global, regional, and national burden of 7 major ADs (RA, IBD, MS, T1DM, RHD, psoriasis, and AA) in older adolescents and young adults from 1990 to 2019, based on data from the GBD 2019 study. The key burden indicators, including incidence, prevalence, mortality, and DALYs, were examined to elucidate spatiotemporal patterns and disparities.

Globally, the ASIR and ASPR of T1DM, RHD, and RA exhibited an upward trend, while AA, IBD, and psoriasis generally showed declining trends. Despite increasing incidence in certain conditions, reductions in mortality and DALYs were observed for several ADs, suggesting potential improvements in early diagnosis, treatment accessibility, and clinical management over the past three decades. This aligns with existing evidence indicating that the onset of ADs frequently occurs in early life stages, with T1DM, IBD, and AA often presenting in childhood or adolescence,^23-26^ while RA, MS, and psoriasis more commonly emerge in young and middle-aged adults.^27-29^ The increasing burden observed in this age group likely reflects both a true rise in disease occurrence and enhanced diagnostic capabilities, particularly in high-resource settings.^30^

At the regional level, the burden of ADs was disproportionately concentrated in high-income areas, particularly Europe and the Americas, where higher ASIRs and ASPRs were recorded. These patterns are consistent with prior studies and may be partially attributed to improved disease surveillance, healthcare infrastructure, and access to diagnostic services in these regions.^31^ Conversely, sub-Saharan Africa and Northern Africa experienced notable declines in RA burden.^32^ Regional differences may also be influenced by environmental and climatic factors, such as ultraviolet exposure and latitude, which affect vitamin D synthesis, a factor implicated in the pathogenesis of RA, MS, and ankylosing spondylitis.^33-35^ Geographic variations in immune function and infection exposure may further contribute to observed disparities.

Country-level differences were also pronounced. For instance, AA burden was markedly high in the United States and parts of Latin America, potentially due to greater psychosocial stress, rising autoimmune predisposition, and improved healthcare access among youth.^36-38^ Brazil showed a high burden of RA, which may relate to rapid urbanization, lifestyle changes, and enhanced diagnostic capabilities of the health system.^39-42^ These country-specific trends underscore the complex interplay between genetic susceptibility, environmental triggers, and social determinants of health in shaping national AD patterns.

Sex-specific disparities were evident across all metrics. Males exhibited higher burdens of T1DM and IBD, reflecting differences in disease phenotype (e.g., Crohn’s disease versus ulcerative colitis), immune response, gut microbiota, and hormonal influences.^43-47^ In contrast, females bore a disproportionate burden across most other ADs, accounting for approximately 78% of cases globally.^48^ Estrogen has been implicated as a key immunomodulatory hormone, enhancing immune activation, and increasing susceptibility to autoimmunity.^49^ The immune-stimulating effects of estrogen, particularly during puberty and reproductive years, may underlie the pronounced female predominance observed across many Ads.^50,51^ These findings emphasize the importance of sex-specific mechanisms in disease development and the need for gender-sensitive prevention and treatment strategies.

Despite these insights, several limitations should be acknowledged. First, the variability in data availability and quality across countries, particularly low- and middle-income regions, may introduce underestimation or modeling biases. Second, although robust statistical models were employed to estimate the disease burden, regions with sparse raw data are more prone to modeling biases. Third, changes in diagnostic criteria, treatment paradigms, and healthcare utilization over time may influence observed trends, complicating direct comparisons across time and regions.

It is also important to consider the evolving landscape of management of ADs. Over the past 3 decades, the introduction of advanced diagnostic methods, biologics, and immunomodulatory therapies has likely altered the clinical course and outcomes of ADs. Simultaneously, greater public awareness and early screening have contributed to rising detection rates, particularly in high-income countries. These dynamic changes underscore the need for future studies to incorporate clinical data on treatment utilization, disease progression, and real-world outcomes to better contextualize epidemiological trends.

In conclusion, this study reveals a significant and growing burden of ADs among older adolescents and young adults between 1990 and 2019. While global trends in T1DM, RHD, and RA indicate increasing incidence and prevalence, other conditions such as AA, IBD, MS, and psoriasis have shown relative declines in disease burden. Persistent disparities across regions and sexes highlight the need for targeted and equitable public health responses. Policymakers and healthcare systems should prioritize early detection, age-specific interventions, and culturally appropriate care models to reduce the burden of ADs in this vulnerable population and to advance health equity.

## Supplementary Materials

Supplementary Material

## Figures and Tables

**Table 1.** Global and Regional Levels of ASIR, ASPR, ASMR, and ASDR of ADs Among Older Adolescent and Young Adults in 2019

	Location	Sex	AA	T1DM	IBD	MS	Psoriasis	RHD	RA
ASIR	Global	Both	493.84 (444.13, 544.49)	9.25 (4.2, 16.65)	4.63 (3.8, 5.59)	1.15 (0.76, 1.65)	44.86 (38.92, 51.47)	62.64 (34.24, 93.77)	7.2 (5.11, 9.7)
		Female	628.46 (563.56, 693.47)	8.27 (3.7, 15.06)	4.3 (3.54, 5.17)	1.58 (1.06, 2.23)	46.14 (40.00, 53.01)	70.16 (39.03, 104.29)	10.38 (7.4, 13.87)
		Male	362.86 (326.53, 399.64)	10.19 (4.68, 18.22)	4.96 (4.05, 6.00)	0.73 (0.46, 1.07)	43.66 (37.87, 50.01)	55.38 (29.66, 83.89)	4.11 (2.86, 5.66)
	African region	Both	457.46 (411.21, 505.76)	9.77 (4.51, 17.47)	0.81 (0.57, 1.06)	0.48 (0.27, 0.76)	28.27 (24.15, 32.71)	113.2 (57.4, 175.18)	4.09 (2.71, 5.83)
		Female	588.65 (527.43, 651.88)	9.56 (4.43, 17.06)	0.78 (0.55, 1.03)	0.64 (0.38, 1.01)	28.85 (24.72, 33.4)	119.61 (60.9, 183.82)	5.54 (3.74, 7.84)
		Male	317.8 (284.83, 351.97)	10 (4.6, 17.96)	0.84 (0.6, 1.11)	0.3 (0.16, 0.5)	27.65 (23.55, 31.99)	106.5 (53.63, 165.68)	2.55 (1.61, 3.78)
	Eastern Mediterranean region	Both	425.99 (381.32, 471.24)	11.06 (4.96, 20.16)	2.26 (1.67, 2.95)	1.67 (1.03, 2.53)	42.77 (37.05, 48.96)	73.24 (41.99, 107.99)	7.49 (5.28, 10.18)
		Female	560.24 (497.99, 621.83)	9.85 (4.38, 18.19)	2.09 (1.54, 2.73)	2.31 (1.46, 3.45)	44.97 (39.03, 51.52)	81.35 (47.23, 118.01)	9.94 (6.93, 13.52)
		Male	299.87 (268.96, 332.86)	12.19 (5.49, 21.95)	2.42 (1.78, 3.17)	1.07 (0.64, 1.63)	40.71 (35.18, 46.72)	65.63 (37.02, 98.19)	5.18 (3.69, 6.96)
	European region	Both	523.78 (470.04, 577.43)	15.86 (7.13, 28.23)	14.77 (12.36, 17.62)	3.75 (2.49, 5.31)	113.18 (98.74, 129)	8.86 (4.91, 13.38)	8.84 (5.94, 12.53)
		Female	599.15 (535.67, 663.01)	14.59 (6.48, 26.06)	14.24 (11.96, 16.99)	5.11 (3.4, 7.18)	113.53 (98.7, 129.69)	9.01 (4.99, 13.67)	13.6 (9.27, 19.01)
		Male	452.02 (404.72, 500.45)	17.07 (7.73, 30.35)	15.28 (12.77, 18.28)	2.46 (1.57, 3.57)	112.92 (98.52, 128.41)	8.73 (4.88, 13.12)	4.31 (2.78, 6.36)
	Region of the Americas	Both	569.25 (513.55, 626.01)	12.46 (5.51, 22.4)	11.55 (9.77, 13.65)	1.94 (1.34, 2.8)	57.76 (49.83, 66.4)	43.58 (23.29, 66.27)	11.42 (8.57, 15.05)
		Female	719.15 (645.71, 792.94)	11.59 (5.08, 21.02)	11.62 (9.85, 13.73)	2.22 (1.55, 3.11)	59.04 (50.8, 68.21)	46.71 (25.24, 71.29)	12.19 (9.26, 16.01)
		Male	420.67 (379.92, 461.46)	13.32 (5.94, 23.83)	11.48 (9.67, 13.59)	17.18 (14.43, 19.93)	56.56 (48.76, 64.66)	40.51 (21.56, 61.67)	108.96 (96.32, 122.44)
	South-East Asia region	Both	456.64 (407.09, 506.44)	8.42 (3.81, 15.32)	0.81 (0.57, 1.08)	0.45 (0.25, 0.73)	33.53 (28.89, 38.57)	76.86 (45.25, 111.37)	6.79 (4.84, 9.08)
		Female	588.16 (521.16, 653.31)	7.04 (3.09, 12.99)	0.8 (0.57, 1.08)	0.57 (0.33, 0.93)	34.53 (29.77, 39.74)	91.31 (54.36, 131.24)	9.57 (6.9, 12.72)
		Male	329.85 (293.85, 364.93)	9.74 (4.48, 17.51)	0.74 (0.52, 1.02)	0.32 (0.17, 0.54)	32.58 (28.07, 37.48)	63.01 (36.03, 93.33)	4.11 (2.86, 5.61)
	Western Pacific region	Both	547.25 (487.67, 606.74)	4.13 (1.81, 7.73)	4.79 (3.82, 5.91)	0.25 (0.14, 0.4)	36.76 (31.78, 42.24)	29.43 (14.87, 46.59)	6.43 (4.56, 8.63)
		Female	707.7 (628.74, 787.92)	3.19 (1.37, 6.11)	3.78 (3.03, 4.67)	0.32 (0.18, 0.51)	40.08 (34.51, 46.04)	30.9 (15.75, 48.76)	9.61 (6.91, 12.73)
		Male	397.36 (352.2, 441.31)	5.01 (2.22, 9.29)	5.73 (4.57, 7.09)	0.18 (0.09, 0.3)	33.72 (29.14, 38.7)	28.09 (14.09, 44.76)	3.47 (2.35, 4.8)
ASPR	Global	Both	269.71 (243.25, 296.46)	244.64 (182.95, 318.51)	27.82 (23.37, 33.05)	8.18 (6.03, 11.26)	335.42 (308.21, 363.98)	771.43 (529.38, 1074.04)	65.45 (50.53, 83.92)
		Female	343.17 (309.57, 377.71)	239.42 (178.22, 311.78)	29.32 (24.64, 34.76)	11.03 (8.24, 15)	361.36 (332.47, 391.96)	856.02 (590.22, 1184.79)	91.78 (71.34, 117.22)
		Male	198.23 (178.4, 218.08)	249.77 (186.89, 325.98)	26.36 (22.06, 31.49)	5.39 (3.92, 7.59)	310.34 (284.74, 337.24)	689.24 (471.42, 965.98)	39.77 (30.28, 51.35)
	African region	Both	250 (225.34, 275.44)	215.09 (158.11, 284.66)	4.48 (3.19, 5.9)	3.59 (2.38, 5.45)	193.31 (175.92, 212.27)	1589.33 (1097.13, 2217.4)	31.68 (22.46, 44.04)
		Female	321.35 (288.6, 355.31)	209.69 (154.2, 278.21)	4.97 (3.54, 6.55)	4.7 (3.14, 7.07)	197.46 (179.78, 216.7)	1661.85 (1150.64, 2312.44)	42.79 (30.71, 58.54)
		Male	174.02 (156.24, 192.05)	220.82 (162.22, 292.3)	3.94 (2.82, 5.19)	2.39 (1.56, 3.72)	188.93 (171.88, 207.56)	1512.38 (1040.4, 2112.22)	19.8 (13.61, 28.4)
	Eastern Mediterranean region	Both	232.78 (209.21, 256.02)	317.25 (235.55, 415.3)	13.48 (10.07, 17.6)	11.82 (8.26, 17.19)	276.03 (252.3, 301.45)	817.72 (555.99, 1135.85)	64.37 (48.36, 84.89)
		Female	305.99 (274.06, 338.19)	305.54 (227.61, 399.2)	14.67 (10.92, 19.24)	16.33 (11.47, 23.6)	298.88 (273.42, 326.18)	898.74 (614.86, 1243.39)	83.89 (62.47, 111.83)
		Male	164.01 (147.02, 181.65)	328.25 (243.19, 430.78)	12.36 (9.22, 16.12)	7.59 (5.18, 11.4)	254.57 (232.1, 278.91)	741.6 (502.28, 1039.1)	46.04 (35.03, 59.64)
	European region	Both	285.86 (257.77, 314.92)	419.05 (312.96, 542.63)	90.99 (77.56, 107.2)	26.81 (19.98, 36.31)	853.81 (784.88, 927.32)	128.5 (90.3, 174.23)	86.62 (64.3, 114.06)
		Female	327.27 (294.31, 361.23)	412.66 (308.08, 534.21)	99.87 (85.03, 117.48)	35.69 (26.8, 48.1)	928.6 (855.57, 1007.25)	137.49 (97.16, 185.67)	129.75 (97.29, 169.67)
		Male	246.46 (221.08, 273.07)	425.17 (316.38, 548.64)	82.51 (69.98, 97.38)	18.33 (13.5, 25.24)	783.03 (717.47, 851.93)	119.95 (83.57, 163.41)	45.5 (32.83, 61.31)
	Region of the Americas	Both	311.06 (281.69, 340.85)	345.13 (262.63, 438.31)	34.02 (28.91, 39.83)	17.91 (14.2, 22.78)	514.13 (475.8, 554.73)	629.95 (437.12, 864.27)	118 (96, 144.8)
		Female	393.13 (354.57, 432.12)	360.01 (273.19, 456.37)	38.4 (32.52, 44.84)	25.46 (20.23, 32.24)	560.12 (519.18, 603.2)	702.97 (492.31, 953.3)	173.07 (141.55, 211.1)
		Male	229.72 (208.71, 251.25)	330.54 (252.24, 421.23)	29.73 (25.21, 34.99)	10.36 (8.11, 13.41)	468.89 (433.26, 507.13)	557.7 (383.91, 773.15)	63.27 (50.66, 79.26)
	South-East Asia region	Both	249.34 (223.83, 276.68)	211.52 (155.38, 281.39)	40.58 (34.17, 48.32)	3.51 (2.32, 5.33)	244.01 (223.11, 266.32)	795.68 (538.55, 1108.39)	58.66 (44.65, 75.4)
		Female	320.94 (286.69, 356.51)	201.82 (148.05, 269.1)	44.52 (37.58, 52.81)	4.48 (3.04, 6.74)	251.29 (229.47, 274.46)	927.06 (631.91, 1288.39)	79.89 (61.06, 102.26)
		Male	180.29 (160.81, 199.92)	220.91 (161.89, 295.46)	36.85 (30.9, 44.16)	2.57 (1.66, 4.02)	237.03 (216.63, 258.66)	669.02 (449.63, 938.13)	38.14 (28.63, 49.55)
	Western Pacific region	Both	298.56 (267.74, 330.58)	140.91 (105.92, 181.26)	29.79 (24.3, 36.44)	2.08 (1.39, 3.16)	266.21 (244.09, 289.75)	441.92 (300.92, 615.03)	60.08 (46.52, 76.12)
		Female	386.32 (344.81, 429.5)	133.38 (100.86, 170.66)	26.57 (21.61, 32.46)	2.61 (1.76, 3.94)	310.71 (285.35, 337.45)	477.45 (327.1, 659.31)	84.37 (65.21, 106.65)
		Male	216.55 (193.83, 240.58)	148.01 (111.31, 190.85)	32.84 (26.84, 39.96)	1.58 (1.02, 2.44)	225.12 (205.63, 245.86)	408.8 (277.67, 574.75)	37.24 (28.3, 47.97)
ASMR	Global	Both	-	0.46 (0.4, 0.52)	0.06 (0.05, 0.08)	0.03 (0.02, 0.04)	-	1.08 (0.94, 1.23)	0.01 (0.01, 0.01)
		Female	-	0.39 (0.29, 0.46)	0.07 (0.05, 0.09)	0.04 (0.03, 0.05)	-	1.02 (0.86, 1.23)	0.01 (0.01, 0.02)
		Male	-	0.53 (0.47, 0.6)	0.06 (0.04, 0.07)	0.02 (0.02, 0.03)	-	1.14 (0.95, 1.35)	0.01 (0.01, 0.01)
	African region	Both	-	0.55 (0.43, 0.68)	0.09 (0.06, 0.13)	0.02 (0.02, 0.03)	-	0.63 (0.5, 0.8)	0.01 (0.01, 0.01)
		Female	-	0.34 (0.22, 0.45)	0.1 (0.06, 0.16)	0.04 (0.02, 0.05)	-	0.61 (0.46, 0.8)	0.01 (0.01, 0.02)
		Male	-	0.78 (0.61, 0.96)	0.08 (0.06, 0.12)	0.01 (0.01, 0.02)	-	0.64 (0.46, 0.87)	0.01 (0, 0.01)
	Eastern Mediterranean region	Both	-	0.63 (0.46, 0.81)	0.06 (0.04, 0.09)	0.05 (0.03, 0.08)	-	2.11 (1.55, 2.77)	0 (0, 0.01)
		Female	-	0.59 (0.31, 0.87)	0.08 (0.04, 0.14)	0.07 (0.03, 0.12)	-	1.97 (1.35, 2.75)	0.01 (0, 0.01)
		Male	-	0.66 (0.48, 0.9)	0.04 (0.02, 0.06)	0.03 (0.02, 0.05)	-	2.23 (1.44, 3.26)	0 (0, 0.01)
	European region	Both	-	0.39 (0.35, 0.43)	0.07 (0.06, 0.08)	0.06 (0.05, 0.11)	-	0.22 (0.19, 0.26)	0.02 (0.01, 0.02)
		Female	-	0.35 (0.3, 0.41)	0.06 (0.05, 0.07)	0.07 (0.05, 0.14)	-	0.22 (0.18, 0.27)	0.02 (0.01, 0.03)
		Male	-	0.42 (0.38, 0.46)	0.07 (0.06, 0.09)	0.05 (0.04, 0.1)	-	0.22 (0.19, 0.26)	0.01 (0.01, 0.02)
	Region of the Americas	Both	-	0.54 (0.42, 0.6)	0.07 (0.06, 0.08)	0.03 (0.02, 0.04)	-	0.21 (0.18, 0.25)	0.02 (0.02, 0.03)
		Female	-	0.48 (0.31, 0.56)	0.07 (0.06, 0.08)	0.04 (0.03, 0.06)	-	0.24 (0.19, 0.31)	0.03 (0.02, 0.05)
		Male	-	0.6 (0.47, 0.66)	0.07 (0.05, 0.08)	0.02 (0.01, 0.03)	-	0.18 (0.16, 0.22)	0.01 (0.01, 0.02)
	South-East Asia region	Both	-	0.5 (0.39, 0.61)	0.07 (0.04, 0.1)	0.02 (0.02, 0.04)	-	2.09 (1.76, 2.47)	0.01 (0, 0.01)
		Female	-	0.45 (0.28, 0.59)	0.08 (0.04, 0.12)	0.03 (0.02, 0.06)	-	1.95 (1.5, 2.51)	0.01 (0, 0.01)
		Male	-	0.54 (0.44, 0.68)	0.06 (0.04, 0.09)	0.02 (0.01, 0.02)	-	2.22 (1.78, 2.76)	0 (0, 0.01)
	Western Pacific region	Both	-	0.24 (0.21, 0.27)	0.02 (0.02, 0.03)	0.02 (0.01, 0.02)	-	0.42 (0.36, 0.49)	0.01 (0.01, 0.02)
		Female	-	0.21 (0.17, 0.25)	0.02 (0.01, 0.02)	0.02 (0.01, 0.03)	-	0.43 (0.35, 0.52)	0.02 (0.01, 0.02)
		Male	-	0.27 (0.22, 0.31)	0.03 (0.02, 0.04)	0.01 (0.01, 0.02)	-	0.41 (0.33, 0.5)	0.01 (0.01, 0.02)
ASDR	Global	Both	8.96 (5.62, 13.56)	43.11 (36.31, 51.37)	8.45 (6.52, 10.57)	4.14 (3.17, 5.4)	30.07 (20.87, 40.69)	108.36 (88.63, 133.67)	9.97 (6.69, 14.17)
		Female	11.36 (7.13, 17.13)	38.51 (29.8, 47.64)	9.23 (6.74, 11.77)	5.49 (4.13, 7.14)	32.15 (22.33, 43.65)	108.49 (86.79, 137.45)	13.79 (9.19, 19.47)
		Male	6.62 (4.11, 10.04)	47.59 (40.97, 56.34)	7.69 (5.93, 9.72)	2.82 (2.12, 3.78)	28.06 (19.42, 37.93)	108.22 (88.03, 133.51)	6.25 (4.19, 8.92)
	African region	Both	8.28 (5.18, 12.6)	48.21 (38.62, 59.12)	6.82 (4.92, 9.25)	2.55 (1.82, 3.34)	17.31 (11.99, 23.72)	117.84 (82.18, 168.59)	5.17 (3.36, 7.51)
		Female	10.61 (6.66, 16.11)	34.4 (24.88, 44.42)	7.62 (4.55, 11.33)	3.57 (2.35, 4.72)	17.55 (11.98, 24.2)	119.66 (81.19, 172.34)	6.96 (4.49, 10.09)
		Male	5.8 (3.59, 8.94)	62.93 (50.79, 76.82)	5.97 (4.25, 8.15)	1.45 (1.04, 2.02)	17.07 (11.68, 23.25)	115.68 (80.64, 164.12)	3.25 (2.06, 4.76)
	Eastern Mediterranean region	Both	7.73 (4.83, 11.69)	58.43 (45.42, 73.63)	6.02 (4.17, 8.41)	6.46 (4.37, 9.15)	24.72 (16.9, 33.83)	178.44 (136.07, 226.71)	9.43 (6.03, 13.71)
		Female	10.12 (6.27, 15.4)	55.74 (35.17, 76.53)	7.74 (4.43, 12.19)	8.72 (5.7, 13.09)	26.54 (17.99, 36.62)	173.01 (126.19, 228.66)	12.19 (7.65, 18.1)
		Male	5.49 (3.36, 8.43)	60.97 (47.06, 78.36)	4.4 (2.97, 6.35)	4.34 (2.88, 6.13)	23 (15.38, 31.52)	183.56 (128.06, 255.61)	6.85 (4.24, 10.09)
	European region	Both	9.5 (5.96, 14.44)	47.21 (38.07, 59.89)	18.39 (13.33, 24.61)	11.21 (8.11, 15.83)	76.37 (53.02, 103.78)	20.85 (17.08, 25.69)	13.28 (8.78, 19.25)
		Female	10.83 (6.82, 16.49)	45.09 (35.8, 58.25)	19.92 (14.25, 26.8)	14.2 (10.04, 20.2)	82.44 (57.52, 112.76)	21.31 (17.24, 26.49)	19.47 (12.8, 28.22)
		Male	8.24 (5.14, 12.52)	49.25 (40.09, 62.13)	16.93 (12.25, 22.67)	8.36 (5.91, 12.56)	70.62 (48.64, 96.45)	20.42 (16.73, 25.02)	7.38 (4.81, 10.79)
	Region of the Americas	Both	10.3 (6.52, 15.5)	53.11 (42.5, 64.11)	15.45 (11.52, 19.99)	6.9 (5.16, 8.94)	45.72 (31.64, 61.55)	43.86 (30.05, 63.28)	17.92 (12.32, 24.93)
		Female	12.98 (8.21, 19.62)	50.5 (37.23, 62.52)	17.37 (12.75, 22.68)	9.39 (6.83, 12.35)	49.44 (34.38, 66.66)	48.98 (33.25, 70.64)	26.01 (17.86, 36.02)
		Male	7.65 (4.81, 11.59)	55.78 (45.28, 66.2)	13.53 (10.18, 17.33)	4.41 (3.3, 5.73)	42.05 (29.11, 56.98)	38.78 (26.61, 56.53)	9.88 (6.68, 13.91)
	South-East Asia region	Both	8.27 (5.16, 12.52)	43.71 (35.11, 53.19)	5.45 (3.59, 7.45)	2.54 (1.87, 3.51)	21.92 (15.01, 30.02)	175.27 (146.30, 209.34)	8.67 (5.69, 12.59)
		Female	10.6 (6.61, 16.04)	40.27 (27.66, 51.55)	6.24 (3.4, 9.09)	3.4 (2.3, 5.14)	22.36 (15.43, 30.56)	172.54 (136.04, 217.19)	11.65 (7.55, 16.95)
		Male	6.02 (3.74, 9.16)	47.05 (38.35, 57.82)	4.7 (3.4, 7.13)	1.7 (1.26, 2.3)	21.49 (14.41, 29.72)	177.97 (143.85, 219.19)	5.8 (3.75, 8.45)
	Western Pacific region	Both	9.98 (6.23, 15.14)	22.9 (19.37, 27.05)	6.26 (4.45, 8.49)	1.61 (1.22, 2.12)	24.13 (16.58, 33.03)	49.5 (38.75, 63.7)	9.48 (6.36, 13.4)
		Female	12.89 (8.08, 19.63)	20.74 (17, 25.18)	5.52 (3.82, 7.61)	2.11 (1.49, 2.82)	28.01 (19.25, 38.34)	51.87 (40.34, 67.44)	12.99 (8.59, 18.35)
		Male	7.26 (4.47, 11.13)	24.96 (20.48, 30.12)	6.97 (4.96, 9.35)	1.15 (0.85, 1.6)	20.55 (13.96, 28.45)	47.27 (35.99, 61.25)	6.18 (4.21, 8.77)

AA, alopecia areata; AAPC, average annual percentage change; ADs, autoimmune diseases; ASDR, Age-standardized Disability-adjusted Life Year; ASMR, Age-standardized Mortality Rate; ASIR, Age-standardized Incidence Rate; ASPR, Age-standardized Prevalence Rate; IBD, inflammatory bowel disease; MS, multiple sclerosis; T1DM, type 1 diabetes mellitus; RHD, rheumatic heart disease; T1DM, type 1 diabetes mellitus.

**Table 2.** Global and Regional Trends of ASIR, ASPR, ASMR, and ASDR of ADs in Older Adolescent and Young Adults from 1990 to 2019

	**Location**	**Sex**	**Year**	AA		T1DM		IBD		MS		Psoriasis		RHD		RA	
				AAPC (95% CI)	*P*	AAPC (95%CI)	*P*	AAPC (95% CI)	*P*	AAPC (95% CI)	*P*	AAPC (95%CI)	*P*	AAPC (95% CI)	*P*	AAPC (95% CI)	*P*
ASIR	Global	Both	1990-1999	−0.2 (-0.2, -0.2)	<.001	1.0 (0.8, 1.1)	<.001	0.3 (0.2, 0.4)	<.001	0.0 (-0.1, 0.1)	.854	-1.0 (-1.0, -0.9)	<.001	1 (1, 1)	<.001	0.4 (0.4, 0.4)	<.001
		Female		−0.2 (-0.2, −0.1)	<.001	1.4 (1.2, 1.5)	<.001	-0.1 (-0.3, 0.0)	.098	0.0 (-0.1, 0.1)	.932	-1.0 (-1.0, -0.9)	<.001	1 (1, 1)	<.001	0.4 (0.4, 0.4)	<.001
		Male		−0.3 (-0.3, -0.3)	<.001	0.7 (0.6, 0.8)	<.001	0.6 (0.6, 0.7)	<.001	0.0 (-0.2, 0.1)	.705	-0.9 (-1.0, -0.9)	<.001	0.9 (0.9, 1)	<.001	0.4 (0.4, 0.4)	<.001
		Both	2000-2009	-0.1 (-0.1, -0.1)	<.001	2.2 (2.1, 2.3)	<.001	-0.3 (-0.4, -0.2)	<.001	0.1 (0.1, 0.2)	<.001	-0.7 (-0.8, -0.7)	<.001	0.2 (0.1, 0.2)	<.001	0.4 (0.4, 0.4)	<.001
		Female		-0.1 (-0.1, -0.1)	<.001	1.9 (1.6, 2.1)	<.001	-0.3 (-0.3, -0.2)	<.001	0.2 (0.2, 0.2)	<.001	-0.7 (-0.8, -0.7)	<.001	0.1 (0, 0.2)	.080	0.4 (0.4, 0.4)	<.001
		Male		-0.2 (-0.2, -0.2)	<.001	2.6 (2.5, 2.7)	<.001	-0.4 (-0.5, -0.3)	<.001	0.1 (0.0, 0.1)	<.001	-0.8 (-0.8, -0.8)	<.001	0.3 (0.1, 0.5)	<.001	0.5 (0.5, 0.5)	<.001
		Both	2010-2019	-0.1 (-0.1, -0.1)	<.001	1.4 (1.2, 1.6)	<.001	-0.5 (-0.6, -0.4)	<.001	0.0 (-0.1, 0.0)	.536	-0.8 (-0.8, -0.7)	<.001	1.1 (1, 1.2)	<.001	0.1 (-0.2, 0.3)	.595
		Female		-0.1 (-0.1, -0.1)	<.001	1.6 (1.4, 1.7)	<.001	-0.5 (-0.6, -0.5)	<.001	0.1 (0.0, 0.1)	.004	-0.8 (-0.8, -0.8)	<.001	1.3 (1.1, 1.4)	<.001	0.0 (-0.2, 0.3)	.964
		Male		-0.1 (-0.1, -0.1)	<.001	1.3 (1.1, 1.5)	<.001	-0.5 (-0.6, -0.4)	<.001	-0.1 (-0.2, -0.1)	<.001	-0.7 (-0.8, -0.7)	<.001	0.9 (0.7, 1.2)	<.001	0.3 (0.3, 0.4)	<.001
	African region	Both	1990-1999	0.0 (0.0, 0.0)	<.001	0.4 (0.4, 0.4)	<.001	0.5 (0.5, 0.5)	<.001	0.2 (0.2, 0.3)	<.001	-0.6 (-0.6, -0.6)	<.001	0.3 (0.2, 0.3)	<.001	-0.1 (-0.1, 0.0)	.004
		Female		0.0 (0.0, 0.0)	<.001	0.4 (0.4, 0.5)	<.001	0.5 (0.4, 0.5)	<.001	0.3 (0.2, 0.3)	<.001	-0.6 (-0.7, -0.6)	<.001	0.3 (0.2, 0.5)	<.001	-0.1 (-0.1, -0.1)	<.001
		Male		0.0 (0.0, 0.0)	<.001	0.4 (0.3, 0.4)	<.001	0.5 (0.4, 0.6)	<.001	0.1 (0.1, 0.2)	<.001	-0.5 (-0.6, -0.5)	<.001	0.2 (0.2, 0.3)	<.001	0.1 (0.0, 0.2)	.033
		Both	2000-2009	0.0 (0.0, 0.0)	<.001	0.2 (0.2, 0.2)	<.001	-0.4 (-0.4, -0.4)	<.001	0.3 (0.2, 0.3)	<.001	-0.5 (-0.5, -0.5)	<.001	0.1 (0.1, 0.2)	<.001	0.0 (0.0, 0.0)	.005
		Female		0.0 (0.0, 0.0)	<.001	0.2 (0.2, 0.3)	<.001	-0.2 (-0.3, -0.2)	<.001	0.3 (0.3, 0.3)	<.001	-0.5 (-0.5, -0.5)	<.001	0.1 (0.1, 0.1)	<.001	-0.1 (-0.1, -0.1)	<.001
		Male		0.0 (0.0, 0.0)	.529	0.2 (0.2, 0.3)	<.001	-0.5 (-0.6, -0.4)	<.001	0.2 (0.1, 0.3)	<.001	-0.5 (-0.5, -0.5)	<.001	0.2 (0.1, 0.2)	<.001	0.2 (0.2, 0.3)	<.001
		Both	2010-2019	0.0 (0.0, 0.0)	<.001	0.4 (0.4, 0.5)	<.001	0.2 (0.2, 0.3)	<.001	-0.4 (-0.4, -0.3)	<.001	-0.9 (-0.9, -0.9)	<.001	0 (0, 0.1)	.067	-0.6 (-0.7, -0.5)	<.001
		Female		0.0 (0.0, 0.0)	<.001	0.4 (0.4, 0.4)	<.001	0.2 (0.1, 0.3)	<.001	-0.4 (-0.4, -0.3)	<.001	-0.8 (-0.9, -0.8)	<.001	0.1 (0.1, 0.1)	<.001	-0.8 (-0.9, -0.6)	<.001
		Male		0.0 (0.0, 0.0)	.007	0.4 (0.4, 0.5)	<.001	0.2 (0.0, 0.3)	.024	-0.4 (-0.4, -0.4)	<.001	-1.0 (-1.0, -0.9)	<.001	0 (-0.1, 0.1)	.717	-0.3 (-0.4, -0.3)	<.001
	Eastern Mediterranean region	Both	1990-1999	0.0 (0.0, 0.0)	<.001	0.9 (0.9, 1.0)	<.001	1.0 (0.9, 1.1)	<.001	-0.3 (-0.3, -0.2)	<.001	-0.8 (-0.8, -0.7)	<.001	0.4 (0.3, 0.4)	<.001	0.1 (0.1, 0.2)	<.001
		Female		0.0 (0.0, 0.0)	.002	1.0 (1.0, 1.0)	<.001	1.0 (0.9, 1.0)	<.001	-0.2 (-0.3, -0.1)	.001	-0.7 (-0.8, -0.7)	<.001	0.4 (0.3, 0.4)	<.001	0.3 (0.2, 0.4)	<.001
		Male		0.0 (0.0, 0.0)	<.001	0.9 (0.9, 0.9)	<.001	1.0 (0.9, 1.2)	<.001	-0.6 (-0.7, -0.5)	<.001	-0.8 (-0.8, -0.7)	<.001	0.4 (0.3, 0.5)	<.001	-0.2 (-0.3, -0.1)	<.001
		Both	2000-2009	0.0 (0.0, 0.0)	<.001	1.7 (1.6, 1.8)	<.001	-0.7 (-0.8, -0.6)	<.001	0.4 (0.4, 0.4)	<.001	-0.9 (-0.9, -0.9)	<.001	-0.1 (-0.1, 0)	<.001	0.4 (0.4, 0.4)	<.001
		Female		0.0 (0.0, 0.0)	.646	1.6 (1.6, 1.6)	<.001	-0.7 (-0.8, -0.6)	<.001	0.4 (0.4, 0.4)	<.001	-0.9 (-0.9, -0.9)	<.001	0.1 (0, 0.1)	.007	0.4 (0.4, 0.4)	<.001
		Male		0.0 (0.0, 0.0)	.004	1.7 (1.6, 1.8)	<.001	-0.7 (-0.8, -0.6)	<.001	0.4 (0.4, 0.5)	<.001	-0.9 (-1.0, -0.9)	<.001	-0.1 (-0.2, -0.1)	<.001	0.5 (0.5, 0.5)	<.001
		Both	2010-2019	0.0 (0.0, 0.0)	.001	2.1 (2.0, 2.1)	<.001	0.2 (0.1, 0.2)	<.001	-0.1 (-0.1, -0.1)	<.001	-0.8 (-0.8, -0.8)	<.001	0.7 (0.7, 0.8)	<.001	1.0 (1.0, 1.1)	<.001
		Female		0.0 (0.0, 0.0)	<.001	2.1 (2.0, 2.1)	<.001	0.2 (0.2, 0.3)	<.001	-0.2 (-0.3, -0.1)	.001	-0.8 (-0.8, -0.8)	<.001	0.8 (0.7, 0.9)	<.001	0.8 (0.8, 0.8)	<.001
		Male		0.0 (0.0, 0.0)	<.001	2.0 (2.0, 2.1)	<.001	0.1 (0.1, 0.2)	<.001	0.0 (0.0, 0.0)	.002	-0.8 (-0.8, -0.7)	<.001	0.6 (0.6, 0.7)	<.001	1.4 (1.3, 1.5)	<.001
	European region	Both	1990-1999	-0.1 (-0.1, -0.1)	<.001	1.7 (1.7, 1.8)	<.001	1.1 (1, 1.2)	<.001	0.5 (0.4, 0.6)	<.001	-0.7 (-0.8, -0.7)	<.001	0.8 (0.7, 0.9)	<.001	0.4 (0.4, 0.5)	<.001
		Female		0.0 (0.0, 0.0)	<.001	1.7 (1.5, 1.8)	<.001	1.0 (0.9, 1.1)	<.001	0.5 (0.4, 0.6)	<.001	-0.8 (-0.8, -0.7)	<.001	1 (0.9, 1)	<.001	0.4 (0.4, 0.5)	<.001
		Male		-0.3 (-0.3, -0.3)	<.001	1.8 (1.7, 1.9)	<.001	1.2 (0.9, 1.5)	<.001	0.4 (0.3, 0.5)	<.001	-0.7 (-0.7, -0.6)	<.001	0.7 (0.6, 0.7)	<.001	0.4 (0.4, 0.5)	<.001
		Both	2000-2009	0.0 (0.0, 0.0)	<.001	2.8 (2.6, 3.0)	<.001	0.1 (0.1, 0.2)	<.001	0.3 (0.3, 0.3)	<.001	-0.4 (-0.5, -0.4)	<.001	1.9 (1.8, 2.1)	<.001	0.6 (0.6, 0.6)	<.001
		Female		0.0 (0.0, 0.0)	<.001	2.5 (2.4, 2.6)	<.001	0.1 (0.1, 0.1)	<.001	0.4 (0.4, 0.5)	<.001	-0.4 (-0.5, -0.3)	<.001	1.8 (1.8, 1.8)	<.001	0.7 (0.7, 0.7)	<.001
		Male		-0.1 (-0.1, -0.1)	<.001	3.0 (2.9, 3.2)	<.001	0.2 (0.1, 0.2)	<.001	0.2 (0.2, 0.3)	<.001	-0.5 (-0.5, -0.5)	<.001	2.1 (2, 2.2)	<.001	0.5 (0.5, 0.5)	<.001
		Both	2010-2019	0.1 (0.1, 0.1)	<.001	1.2 (1.1, 1.3)	<.001	0.8 (0.8, 0.9)	<.001	0.6 (0.6, 0.7)	<.001	0.2 (0.2, 0.3)	<.001	0.4 (0.3, 0.5)	<.001	0.5 (0.5, 0.5)	<.001
		Female		-0.1 (-0.1, -0.1)	<.001	1.5 (1.4, 1.6)	<.001	0.8 (0.8, 0.9)	<.001	0.9 (0.7, 1.0)	<.001	0.1 (0.1, 0.2)	<.001	0.4 (0.3, 0.5)	<.001	0.6 (0.5, 0.6)	<.001
		Male		0.3 (0.2, 0.3)	<.001	0.9 (0.8, 1.0)	<.001	0.9 (0.8, 0.9)	<.001	0.3 (0.2, 0.3)	<.001	0.3 (0.2, 0.3)	<.001	0.4 (0.3, 0.5)	<.001	0.5 (0.4, 0.5)	<.001
	Region of the Americas	Both	1990-1999	-0.5 (-0.6, -0.5)	<.001	0.3 (-0.2, 0.7)	.218	-3.0 (-3.2, -2.9)	<.001	0.4 (0.3, 0.4)	<.001	-0.5 (-0.5, -0.5)	<.001	0.5 (0.4, 0.6)	<.001	1.1 (1.0, 1.2)	<.001
		Female		-0.3 (-0.3, -0.2)	<.001	0.6 (0.3, 0.9)	<.001	-2.9 (-3.1, -2.8)	<.001	0.4 (0.4, 0.5)	<.001	-0.5 (-0.5, -0.5)	<.001	0.6 (0.5, 0.6)	<.001	0.7 (0.6, 0.8)	<.001
		Male		-0.9 (-0.9, -0.8)	<.001	0.1 (0, 0.2)	.023	-3.1 (-3.3, -2.9)	<.001	-0.1 (-0.2, -0.1)	<.001	-0.5 (-0.6, -0.4)	<.001	0.4 (0.4, 0.5)	<.001	0.2 (0.2, 0.3)	<.001
		Both	2000-2009	0.0 (-0.1, 0.0)	<.001	0.8 (0.5, 1.0)	<.001	0.4 (0.4, 0.5)	<.001	0.4 (0.3, 0.4)	<.001	-0.5 (-0.6, -0.5)	<.001	0.0 (0.0, 0.0)	.175	0.5 (0.4, 0.5)	<.001
		Female		0.0 (0.0, 0.0)	<.001	0.5 (0.1, 0.9)	.022	0.5 (0.4, 0.7)	<.001	0.4 (0.4, 0.5)	<.001	-0.5 (-0.5, -0.5)	<.001	0 (-0.1, 0)	<.001	0.7 (0.6, 0.8)	<.001
		Male		0.0 (-0.1, 0.0)	<.001	1.1 (1, 1.2)	<.001	0.4 (0.4, 0.5)	<.001	-0.1 (-0.2, -0.1)	<.001	-0.6 (-0.7, -0.5)	<.001	0.1 (0, 0.1)	.032	0.2 (0.2, 0.3)	<.001
		Both	2010-2019	0.0 (0.0, 0.0)	.001	0.8 (0.6, 1.0)	<.001	-0.2 (-0.3, -0.2)	<.001	-3.0 (-3.9, -2.1)	<.001	-0.3 (-0.3, -0.3)	<.001	0 (0, 0.1)	.158	-0.2 (-0.3, 0.0)	.079
		Female		0.0 (0.0, 0.0)	<.001	1.0 (0.6, 1.4)	<.001	-0.3 (-0.3, -0.2)	<.001	-4.9 (-5.9, -4.0)	<.001	-0.3 (-0.3, -0.3)	<.001	0 (-0.1, 0.1)	.764	-3.2 (-3.9, -2.5)	<.001
		Male		0.0 (0.0, 0.0)	.235	0.7 (0.6, 0.8)	<.001	-0.2 (-0.2, -0.1)	<.001	29.4 (28.7, 30.1)	<.001	-0.3 (-0.4, -0.3)	<.001	0.1 (0, 0.1)	.015	37.3 (36.5, 38.1)	<.001
	South-East Asia region	Both	1990-1999	0.0 (0.0, 0.0)	<.001	-0.5 (-0.7, -0.4)	<.001	0.8 (0.6, 1.1)	<.001	0.4 (0.4, 0.5)	<.001	0.1 (0.0, 0.1)	<.001	0.8 (0.7, 0.9)	<.001	0.5 (0.4, 0.5)	<.001
		Female		-0.1 (-0.1, -0.1)	<.001	0.1 (-0.1, 0.3)	.188	0.9 (0.7, 1.1)	<.001	0.4 (0.3, 0.5)	<.001	0.1 (0.1, 0.1)	<.001	0.7 (0.5, 0.9)	<.001	0.5 (0.4, 0.5)	<.001
		Male		-0.1 (-0.1, 0.0)	<.001	-1.0 (-1.2, -0.8)	<.001	0.7 (0.4, 1.0)	<.001	0.4 (0.4, 0.5)	<.001	0.0 (0.0, 0.1)	<.001	0.9 (0.8, 0.9)	<.001	0.4 (0.4, 0.4)	<.001
		Both	2000-2009	-0.1 (-0.1, -0.1)	<.001	4.1 (3.7, 4.5)	<.001	0.8 (0.6, 1.1)	<.001	0.6 (0.5, 0.6)	<.001	-0.1 (-0.1, -0.1)	<.001	-0.8 (-1.4, -0.2)	.007	0.6 (0.5, 0.6)	<.001
		Female		-0.1 (-0.1, -0.1)	<.001	3.6 (3.2, 4.0)	<.001	0.9 (0.7, 1.1)	<.001	0.7 (0.6, 0.7)	<.001	-0.1 (-0.1, -0.1)	<.001	-1.0 (-1.5, -0.5)	<.001	0.6 (0.6, 0.6)	<.001
		Male		-0.2 (-0.2, -0.1)	<.001	4.3 (3.9, 4.7)	<.001	0.7 (0.4, 1.0)	<.001	0.5 (0.5, 0.5)	<.001	-0.1 (-0.1, -0.1)	<.001	-0.2 (-0.3, -0.1)	<.001	0.7 (0.7, 0.7)	<0.001
		Both	2010-2019	-0.1 (-0.1, -0.1)	<.001	1.7 (1.6, 1.7)	<.001	-4.5 (-6.1, -2.8)	<.001	0.4 (0.4, 0.4)	<.001	-0.5 (-0.5, -0.4)	<.001	0.4 (-0.5, 1.2)	.370	0.8 (0.7, 0.8)	<.001
		Female		-0.1 (-0.1, -0.1)	<.001	1.5 (1.4, 1.5)	<.001	-3.5 (-4.7, -2.2)	<.001	0.4 (0.4, 0.4)	<.001	-0.4 (-0.5, -0.4)	<.001	0.5 (-0.1, 1.2)	.104	0.7 (0.7, 0.8)	<.001
		Male		-0.1 (-0.1, -0.1)	<.001	1.8 (1.7, 1.9)	<.001	-6.2 (-8.6, -3.7)	<.001	0.4 (0.3, 0.4)	<.001	-0.5 (-0.5, -0.4)	<.001	0.2 (0.0, 0.4)	.073	0.8 (0.8, 0.8)	<.001
	Western Pacific region	Both	1990-1999	0.0 (0.0, 0.0)	<.001	1.5 (1.3, 1.7)	<.001	8.3 (8.1, 8.5)	<.001	0.6 (0.4, 0.8)	<.001	-1.1 (-1.1, -1)	<.001	-1.1 (-1.2, -1.1)	<.001	0.4 (0.4, 0.4)	<.001
		Female		0.0 (0.0, 0.0)	<.001	3.2 (3.1, 3.4)	<.001	7.7 (7.5, 7.9)	<.001	0.7 (0.5, 0.9)	<.001	-1 (-1.1, -1)	<.001	-1.2 (-1.3, -1.2)	<.001	0.4 (0.3, 0.4)	<.001
		Male		0.1 (0.1, 0.1)	<.001	0.5 (0.3, 0.7)	<.001	8.7 (8.5, 8.9)	<.001	0.4 (0.2, 0.6)	<.001	-1.1 (-1.1, -1.1)	<.001	-1.1 (-1.2, -0.9)	<.001	0.3 (0.3, 0.3)	<.001
		Both	2000-2009	0.0 (0.0, 0.0)	<.001	4.5 (4.1, 4.9)	<.001	0.0 (-0.2, 0.2)	.959	0.0 (-0.1, 0.0)	.283	-0.7 (-0.8, -0.7)	<.001	-0.4 (-0.5, -0.3)	<.001	0.7 (0.6, 0.7)	<.001
		Female		0.0 (0.0, 0.0)	<.001	2.8 (2.5, 3.0)	<.001	0.2 (-0.1, 0.4)	.146	-0.1 (-0.2, 0.0)	.136	-0.6 (-0.7, -0.6)	<.001	-0.2 (-0.3, -0.1)	<.001	0.7 (0.6, 0.7)	<.001
		Male		0.0 (0.0, 0.0)	<.001	5.5 (5.0, 6.1)	<.001	-0.1 (-0.3, 0.2)	.482	0.0 (-0.1, 0.1)	.438	-0.8 (-0.8, -0.8)	<.001	-0.5 (-0.7, -0.3)	<.001	0.6 (0.6, 0.6)	<.001
		Both	2010-2019	0.0 (0.0, 0.0)	<.001	1.2 (0.9, 1.4)	<.001	1.1 (1, 1.2)	<.001	1.1 (0.5, 1.6)	<.001	-1 (-1, -0.9)	<.001	0.1 (0.0, 0.1)	.135	-0.7 (-1.1, -0.4)	<.001
		Female		0.0 (0.0, 0.0)	.034	1.5 (1.3, 1.6)	<.001	1.2 (1.1, 1.3)	<.001	1.2 (0.7, 1.8)	<.001	-1 (-1, -0.9)	<.001	0.2 (0.2, 0.3)	<.001	-0.7 (-1, -0.4)	<.001
		Male		0.1 (0.1, 0.2)	<.001	0.9 (0.6, 1.2)	<.001	1.0 (0.9, 1.1)	<.001	0.9 (0.6, 1.3)	<.001	-0.9 (-1, -0.9)	<.001	-0.1 (-0.2, 0.0)	.023	-0.6 (-0.7, -0.4)	<.001
ASPR	Global	Both	1990-1999	-0.2 (-0.2, -0.2)	<.001	0.5 (0.5, 0.6)	<.001	0.7 (0.6, 0.9)	<.001	-0.3 (-0.4, -0.1)	<.001	-1 (-1, -0.9)	<.001	0.0 (-0.1, 0.0)	.836	0.2 (0.2, 0.3)	<.001
		Female		-0.1 (-0.2, -0.1)	<.001	0.6 (0.4, 0.7)	<.001	0.2 (0.1, 0.3)	<.001	-0.3 (-0.5, -0.1)	.001	-1 (-1, -1)	<.001	0.0 (-0.1, 0.1)	.686	0.2 (0.2, 0.2)	<.001
		Male		-0.3 (-0.3, -0.3)	<.001	0.5 (0.5, 0.6)	<.001	1.4 (1.2, 1.5)	<.001	-0.3 (-0.5, -0.2)	<.001	-0.9 (-0.9, -0.9)	<.001	0.0 (-0.1, 0.0)	.085	0.3 (0.3, 0.3)	<.001
		Both	2000-2009	-0.1 (-0.1, -0.1)	<.001	1.0 (1.0, 1.0)	<.001	-0.5 (-0.5, -0.5)	<.001	0.1 (0.1, 0.2)	<.001	-1 (-1, -1)	<.001	1.1 (1.1, 1.2)	<.001	0.4 (0.4, 0.5)	<.001
		Female		-0.1 (-0.1, -0.1)	<.001	0.9 (0.9, 0.9)	<.001	-0.5 (-0.5, -0.4)	<.001	0.2 (0.2, 0.2)	<.001	-1 (-1, -1)	<.001	1.1 (1.1, 1.1)	<.001	0.4 (0.4, 0.4)	<.001
		Male		-0.2 (-0.2, -0.2)	<.001	1.1 (1.1, 1.2)	<.001	-0.6 (-0.6, -0.5)	<.001	0.1 (0.0, 0.1)	<.001	-0.9 (-0.9, -0.9)	<.001	1.2 (1.1, 1.2)	<.001	0.5 (0.5, 0.5)	<.001
		Both	2010-2019	-0.1 (-0.1, -0.1)	<.001	1.1 (1.0, 1.2)	<.001	-0.8 (-0.9, -0.8)	<.001	-0.1 (-0.2, -0.1)	<.001	-0.9 (-0.9, -0.8)	<.001	0.9 (0.8, 1.1)	<.001	0.0 (-0.1, 0.1)	.830
		Female		-0.1 (-0.1, -0.1)	<.001	1.1 (1.1, 1.2)	<.001	-0.8 (-0.8, -0.8)	<.001	-0.1 (-0.1, 0.0)	.028	-0.9 (-1, -0.9)	<.001	1.0 (0.9, 1.1)	<.001	0.0 (-0.1, 0.0)	.410
		Male		-0.1 (-0.1, -0.1)	<.001	1.1 (1.0, 1.2)	<.001	-0.8 (-0.9, -0.8)	<.001	-0.2 (-0.2, -0.1)	<.001	-0.8 (-0.9, -0.7)	<.001	0.9 (0.7, 1.0)	<.001	0.2 (0.1, 0.3)	<.001
	African region	Both	1990-1999	0.0 (0.0, 0.0)	<.001	0.2 (0.2, 0.2)	<.001	0.2 (0.2, 0.2)	<.001	0.2 (0.2, 0.2)	<.001	-0.6 (-0.6, -0.6)	<.001	0.3 (0.2, 0.3)	<.001	0.1 (0.1, 0.1)	<.001
		Female		0.0 (0.0, 0.0)	<.001	0.2 (0.2, 0.2)	<.001	0.2 (0.2, 0.2)	<.001	0.2 (0.2, 0.2)	<.001	-0.7 (-0.7, -0.6)	<.001	0.3 (0.2, 0.5)	<.001	0.1 (0.0, 0.1)	<.001
		Male		0.0 (0.0, 0.0)	<.001	0.2 (0.2, 0.2)	<.001	0.2 (0.2, 0.3)	<.001	0.1 (0.1, 0.2)	<.001	-0.6 (-0.6, -0.5)	<.001	0.2 (0.2, 0.3)	<.001	0.2 (0.1, 0.2)	<.001
		Both	2000-2009	0.0 (0.0, 0.0)	<.001	0.2 (0.2, 0.2)	<.001	0.2 (0.1, 0.2)	<.001	0.2 (0.2, 0.3)	<.001	-0.5 (-0.5, -0.5)	<.001	0.2 (0.2, 0.3)	<.001	0.1 (0.1, 0.1)	<.001
		Female		0.0 (0.0, 0.0)	.002	0.2 (0.2, 0.2)	<.001	0.3 (0.3, 0.3)	<.001	0.3 (0.3, 0.3)	<.001	-0.5 (-0.6, -0.5)	<.001	0.2 (0.1, 0.3)	<.001	0.1 (0.0, 0.1)	<.001
		Male		0.0 (0.0, 0.0)	.540	0.1 (0.1, 0.2)	<.001	0.0 (-0.1, 0.1)	.761	0.2 (0.1, 0.2)	<.001	-0.5 (-0.5, -0.5)	<.001	0.3 (0.3, 0.3)	<.001	0.3 (0.3, 0.3)	<.001
		Both	2010-2019	0.0 (0.0, 0.0)	<.001	0.3 (0.3, 0.3)	<.001	-0.2 (-0.3, -0.2)	<.001	-0.4 (-0.4, -0.4)	<.001	-1 (-1, -0.9)	<.001	0.2 (0.1, 0.3)	.005	-0.4 (-0.6, -0.1)	.002
		Female		0.0 (0.0, 0.0)	<.001	0.3 (0.3, 0.3)	<.001	-0.2 (-0.3, -0.2)	<.001	-0.4 (-0.4, -0.4)	<.001	-0.9 (-1, -0.9)	<.001	0.2 (0.1, 0.3)	.001	-0.5 (-0.7, -0.4)	<.001
		Male		0.0 (0.0, 0.0)	<.001	0.4 (0.3, 0.4)	<.001	-0.3 (-0.3, -0.2)	<.001	-0.3 (-0.4, -0.3)	<.001	-1 (-1.1, -0.9)	<.001	0.1 (0.0, 0.2)	.006	-0.1 (-0.1, 0.0)	.001
	Eastern Mediterranean region	Both	1990-1999	0.0 (0.0, 0.0)	<.001	1.2 (1.1, 1.2)	<.001	0.7 (0.6, 0.8)	<.001	-0.1 (-0.2, -0.1)	<.001	-0.9 (-0.9, -0.8)	<.001	0.7 (0.7, 0.8)	<.001	0.1 (0.1, 0.2)	<.001
		Female		0.0 (0.0, 0.0)	<.001	1.2 (1.2, 1.3)	<.001	0.5 (0.5, 0.6)	<.001	-0.1 (-0.2, 0.0)	.162	-0.9 (-0.9, -0.8)	<.001	0.7 (0.6, 0.8)	<.001	0.3 (0.2, 0.4)	<.001
		Male		0.0 (0.0, 0.0)	<.001	1.1 (1.1, 1.2)	<.001	0.9 (0.7, 1.0)	<.001	-0.4 (-0.6, -0.3)	<.001	-0.9 (-1, -0.8)	<.001	0.9 (0.8, 1.0)	<.001	-0.2 (-0.3, -0.1)	.001
		Both	2000-2009	0.0 (0.0, 0.0)	<.001	1.6 (1.6, 1.7)	<.001	0 (-0.1, 0.1)	.969	0.4 (0.4, 0.4)	<.001	-1.1 (-1.1, -1)	<.001	-0.3 (-0.4, -0.3)	<.001	0.4 (0.4, 0.5)	<.001
		Female		0.0 (0.0, 0.0)	.180	1.6 (1.5, 1.7)	<.001	0.1 (0, 0.1)	.002	0.4 (0.4, 0.4)	<.001	-1 (-1.1, -1)	<.001	-0.3 (-0.4, -0.2)	<.001	0.5 (0.4, 0.5)	<.001
		Male		0.0 (0.0, 0.0)	.002	1.7 (1.6, 1.7)	<.001	0.0 (-0.1, 0.0)	.311	0.4 (0.4, 0.5)	<.001	-1.1 (-1.2, -1.1)	<.001	-0.4 (-0.5, -0.3)	<.001	0.4 (0.3, 0.5)	<.001
		Both	2010-2019	0.0 (0.0, 0.0)	<.001	2.1 (2.1, 2.2)	<.001	-0.3 (-0.3, -0.2)	<.001	-0.2 (-0.2, -0.1)	<.001	-0.8 (-0.8, -0.8)	<.001	0.4 (0.3, 0.5)	<.001	0.9 (0.9, 1.0)	<.001
		Female		0.0 (0.0, 0.0)	<.001	2.1 (2.1, 2.2)	<.001	-0.3 (-0.3, -0.3)	<.001	-0.3 (-0.3, -0.2)	<.001	-0.9 (-0.9, -0.8)	<.001	0.5 (0.4, 0.6)	<.001	0.7 (0.6, 0.7)	<.001
		Male		0.0 (0.0, 0.0)	<.001	2.2 (2.1, 2.2)	<.001	-0.2 (-0.3, -0.2)	<.001	0.0 (-0.1, 0.0)	.350	-0.7 (-0.8, -0.7)	<.001	0.2 (0.2, 0.3)	<.001	1.3 (1.2, 1.4)	<.001
	European region	Both	1990-1999	-0.1 (-0.1, -0.1)	<.001	1.5 (1.5, 1.6)	<.001	1.4 (1.2, 1.7)	<.001	0.5 (0.3, 0.6)	<.001	-0.9 (-0.9, -0.9)	<.001	0.6 (0.5, 0.6)	<.001	0.3 (0.3, 0.4)	<.001
		Female		0.0 (0.0, 0.0)	<.001	1.6 (1.5, 1.6)	<.001	1.3 (1.1, 1.5)	<.001	0.5 (0.4, 0.7)	<.001	-0.9 (-0.9, -0.8)	<.001	0.6 (0.6, 0.7)	<.001	0.3 (0.3, 0.3)	<.001
		Male		-0.3 (-0.3, -0.3)	<.001	1.5 (1.4, 1.6)	<.001	1.6 (1.3, 1.8)	<.001	0.4 (0.3, 0.4)	<.001	-0.9 (-1, -0.9)	<.001	0.5 (0.5, 0.6)	<.001	0.3 (0.3, 0.4)	<.001
		Both	2000-2009	0.0 (0.0, 0.0)	<.001	1.9 (1.9, 2.0)	<.001	-0.1 (-0.1, 0.0)	.010	0.3 (0.2, 0.3)	<.001	-0.6 (-0.6, -0.5)	<.001	2.0 (1.9, 2.1)	<.001	0.5 (0.4, 0.6)	<.001
		Female		0.0 (0.0, 0.0)	<.001	1.7 (1.7, 1.7)	<.001	-0.1 (-0.2, -0.1)	<.001	0.3 (0.3, 0.3)	<.001	-0.6 (-0.6, -0.5)	<.001	1.9 (1.8, 2.0)	<.001	0.6 (0.5, 0.6)	<.001
		Male		-0.1 (-0.1, -0.1)	<.001	2.1 (2.1, 2.2)	<.001	0.0 (-0.1, 0.0)	.253	0.2 (0.2, 0.3)	<.001	-0.6 (-0.6, -0.5)	<.001	2.1 (2.0, 2.2)	<.001	0.5 (0.4, 0.5)	<.001
		Both	2010-2019	0.1 (0.1, 0.1)	<.001	1.4 (1.3, 1.5)	<.001	0.5 (0.4, 0.5)	<.001	0.5 (0.5, 0.5)	<.001	0.1 (0, 0.1)	.008	0.9 (0.8, 0.9)	<.001	0.7 (0.6, 0.7)	<.001
		Female		-0.1 (-0.1, -0.1)	<.001	1.5 (1.4, 1.5)	<.001	0.4 (0.4, 0.5)	<.001	0.7 (0.6, 0.7)	<.001	0 (0, 0.1)	.220	0.9 (0.8, 1.0)	<.001	0.7 (0.6, 0.8)	<.001
		Male		0.3 (0.2, 0.3)	<.001	1.3 (1.2, 1.4)	<.001	0.5 (0.3, 0.8)	<.001	0.3 (0.2, 0.3)	<.001	0.1 (0.1, 0.2)	<.001	0.9 (0.8, 0.9)	<.001	0.7 (0.6, 0.7)	<.001
	Region of the Americas	Both	1990-1999	-0.5 (-0.6, -0.5)	<.001	-0.5 (-0.6, -0.4)	<.001	-1.9 (-2.2, -1.6)	<.001	-0.4 (-0.5, -0.4)	<.001	-0.2 (-0.2, -0.1)	<.001	0.6 (0.5, 0.6)	<.001	0.7 (0.6, 0.8)	<.001
		Female		-0.3 (-0.3, -0.2)	<.001	-0.4 (-0.5, -0.3)	<.001	-2.0 (-2.3, -1.7)	<.001	-0.4 (-0.5, -0.4)	<.001	-0.2 (-0.3, -0.1)	<.001	0.6 (0.6, 0.6)	<.001	0.7 (0.6, 0.8)	<.001
		Male		-0.9 (-0.9, -0.9)	<.001	-0.6 (-0.6, -0.5)	<.001	-1.7 (-2.0, -1.4)	<.001	-0.4 (-0.4, -0.3)	<.001	-0.2 (-0.2, -0.1)	<.001	0.5 (0.4, 0.5)	<.001	0.8 (0.7, 0.9)	<.001
		Both	2000-2009	0.0(-0.1, 0.0)	<.001	0.6 (0.6, 0.7)	<.001	-6.9 (-8.4, -5.3)	<.001	0.0 (-0.1, 0.1)	.546	-1.3 (-1.3, -1.2)	<.001	0.1 (0.1, 0.1)	<.001	0.6 (0.6, 0.7)	<.001
		Female		0.0 (0.0, 0.0)	<.001	0.5 (0.5, 0.6)	<.001	-6.9 (-8.4, -5.4)	<.001	0.1 (0.0, 0.2)	.053	-1.3 (-1.4, -1.3)	<.001	0.1 (0.1, 0.2)	<.001	0.7 (0.6, 0.7)	<.001
		Male		0.0 (-0.1, 0.0)	<.001	0.7 (0.6, 0.9)	<.001	-6.7 (-8.3, -5.1)	<.001	-0.1 (-0.2, 0.0)	.022	-1.2 (-1.3, -1.2)	<.001	0.1 (0.0, 0.1)	<.001	0.7 (0.6, 0.8)	<.001
		Both	2010-2019	0.0 (0.0, 0.0)	.002	0.5 (0.3, 0.6)	<.001	-1.0 (-1.3, -0.8)	<.001	0.1 (0.1, 0.1)	<.001	-0.4 (-0.5, -0.4)	<.001	0.0 (0.0, 0.1)	<.001	0.3 (0.2, 0.3)	<.001
		Female		0.0 (0.0, 0.0)	<.001	0.6 (0.5, 0.6)	<.001	-0.9 (-1.2, -0.7)	<.001	0.1 (0.1, 0.1)	<.001	-0.4 (-0.5, -0.3)	<.001	0.0 (0.0, 0.0)	.592	0.3 (0.3, 0.4)	<.001
		Male		0.0 (0.0, 0.0)	.688	0.3 (0.2, 0.4)	<.001	-1.2 (-1.5, -0.9)	<.001	0.1 (0.0, 0.1)	<.001	-0.4 (-0.5, -0.4)	<.001	0.1 (0.0, 0.1)	<.001	0.3 (0.2, 0.3)	<.001
	South-East Asia region	Both	1990-1999	0.0 (0.0, 0.0)	<.001	0.1 (0.1, 0.1)	<.001	0.4 (0.4, 0.5)	<.001	0.4 (0.4, 0.5)	<.001	0.3 (0.3, 0.3)	<.001	-0.6 (-0.6, -0.5)	<.001	0.5 (0.4, 0.5)	<.001
		Female		-0.1 (-0.1, -0.1)	<.001	0.2 (0.1, 0.2)	<.001	0.3 (0.2, 0.4)	<.001	0.4 (0.4, 0.5)	<.001	0.3 (0.3, 0.3)	<.001	-0.7 (-0.7, -0.7)	<.001	0.5 (0.5, 0.5)	<.001
		Male		-0.1 (-0.1, 0.0)	<.001	0.1 (0, 0.1)	<.001	0.6 (0.5, 0.7)	<.001	0.4 (0.4, 0.4)	<.001	0.3 (0.3, 0.3)	<.001	-0.5 (-0.6, -0.4)	<.001	0.4 (0.4, 0.4)	<.001
		Both	2000-2009	-0.1 (-0.1, -0.1)	<.001	0.9 (0.9, 0.9)	<.001	0.4 (0.4, 0.5)	<.001	0.5 (0.4, 0.5)	<.001	0.0 (0.0, 0.0)	.088	1.2 (1.2, 1.2)	<.001	0.6 (0.6, 0.6)	<.001
		Female		-0.1 (-0.1, -0.1)	<.001	0.9 (0.9, 1)	<.001	0.3 (0.2, 0.4)	<.001	0.5 (0.5, 0.6)	<.001	0.0 (0.0, 0.0)	.702	1.1 (1.0, 1.1)	<.001	0.6 (0.6, 0.7)	<.001
		Male		-0.1 (-0.2, -0.1)	<.001	0.9 (0.9, 0.9)	<.001	0.6 (0.5, 0.7)	<.001	0.4 (0.4, 0.4)	<.001	0 (0, 0.1)	<.001	1.3 (1.2, 1.3)	<.001	0.6 (0.6, 0.7)	<.001
		Both	2010-2019	-0.1 (-0.1, -0.1)	<.001	0.9 (0.9, 1)	<.001	7.7 (7.1, 8.3)	<.001	0.3 (0.2, 0.3)	<.001	-0.4 (-0.5, -0.3)	<.001	0.2 (0.0, 0.3)	.011	0.8 (0.8, 0.8)	<.001
		Female		-0.1 (-0.1, -0.1)	<.001	0.9 (0.8, 0.9)	<.001	7.9 (7.3, 8.5)	<.001	0.2 (0.2, 0.3)	<.001	-0.4 (-0.5, -0.3)	<.001	0.3 (0.1, 0.5)	<.001	0.8 (0.7, 0.8)	<.001
		Male		-0.1 (-0.1, -0.1)	<.001	1.0 (1.0, 1.0)	<.001	7.5 (6.8, 8.1)	<.001	0.3 (0.3, 0.4)	<.001	-0.4 (-0.5, -0.3)	<.001	0.0 (-0.4, 0.4)	.916	0.8 (0.8, 0.8)	<.001
	Western Pacific region	Both	1990-1999	0.0 (0.0, 0.0)	<.001	0.5 (0.4, 0.6)	<.001	8.9 (8.6, 9.3)	<.001	0.3 (0.0, 0.5)	.023	-1.1 (-1.2, -1.1)	<.001	-3.2 (-3.2, -3.1)	<.001	0.5 (0.4, 0.5)	<.001
		Female		0.0 (0.0, 0.0)	.001	0.7 (0.6, 0.7)	<.001	8.2 (7.8, 8.5)	<.001	0.3 (0.1, 0.6)	.012	-1.1 (-1.1, -1)	<.001	-3.2 (-3.4, -3.0)	<.001	0.4 (0.4, 0.5)	<.001
		Male		0.1 (0.1, 0.1)	<.001	0.3 (0.2, 0.5)	<.001	9.6 (9.2, 10.0)	<.001	0.1 (0.0, 0.2)	.228	-1.3 (-1.3, -1.2)	<.001	-3.2 (-3.3, -3.1)	<.001	0.4 (0.4, 0.4)	<.001
		Both	2000-2009	0.0 (0.0, 0.0)	<.001	0.7 (0.6, 0.9)	<.001	-0.1 (-0.3, 0.2)	.622	0.1 (0.0, 0.3)	.125	-0.9 (-1, -0.9)	<.001	1.1 (0.9, 1.2)	<.001	0.8 (0.7, 0.8)	<.001
		Female		0.0 (0.0, 0.0)	<.001	0.4 (0.4, 0.5)	<.001	-0.1 (-0.3, 0.1)	.216	0.1 (-0.1, 0.3)	.273	-0.9 (-0.9, -0.9)	<.001	1.4 (1.3, 1.5)	<.001	0.7 (0.7, 0.7)	<.001
		Male		0.0 (0.0, 0.0)	<.001	1.1 (0.9, 1.2)	<.001	0.0 (-0.2, 0.3)	.880	0.1 (0.0, 0.2)	.002	-0.9 (-1, -0.9)	<.001	0.9 (0.7, 1.1)	<.001	0.7 (0.7, 0.8)	<.001
		Both	2010-2019	0.0 (0.0, 0.0)	<.001	1.8 (1.6, 1.9)	<.001	0.3 (0.1, 0.4)	<.001	0.8 (0.5, 1.2)	<.001	-1 (-1, -1)	<.001	0.2 (0.1, 0.2)	<.001	-0.8 (-1.2, -0.4)	<.001
		Female		0.0 (0.0, 0.0)	.851	1.8 (1.7, 2.0)	<.001	0.2 (0.1, 0.3)	.004	0.9 (0.5, 1.3)	<.001	-1 (-1, -0.9)	<.001	0.3 (0.3, 0.4)	<.001	-0.8 (-1.1, -0.4)	<.001
		Male		0.1 (0.1, 0.2)	<.001	1.6 (1.3, 1.9)	<.001	0.3 (0.1, 0.5)	<.001	0.9 (0.6, 1.1)	<.001	-1 (-1, -0.9)	<.001	0.1 (0.0, 0.1)	.005	-0.5 (-0.6, -0.4)	<.001
ASMR	Global	Both	1990-1999	-	-	1.3 (1.1, 1.5)	<.001	-0.3 (-0.4, -0.2)	<.001	0.9 (0.6, 1.1)	<.001	-	-	-0.5 (-1.2, 0.2)	.126	-1.3 (-1.8, -0.7)	<.001
		Female		-	-	1.1 (0.8, 1.4)	<.001	0.2 (0.0, 0.4)	.025	1.3 (1.0, 1.7)	<.001	-	-	-0.9 (-1.1, -0.7)	<.001	-0.9 (-1.2, -0.7)	<.001
		Male		-	-	1.3 (1.1, 1.4)	<.001	-1.0 (-1.2, -0.7)	<.001	0.0 (-0.2, 0.3)	.889	-	-	-0.3 (-1.1, 0.5)	.442	-1.8 (-2.7, -0.9)	<.001
		Both	2000-2009	-	-	-1.6 (-1.7, -1.6)	<.001	-2.3 (-2.8, -1.8)	<.001	-1.7 (-2, -1.3)	<.001	-	-	-3.0 (-3.3, -2.6)	<.001	-2.7 (-3.0, -2.5)	<.001
		Female		-	-	-2.5 (-2.6, -2.3)	<.001	-2.5 (-3.3, -1.6)	<.001	-1.7 (-2.2, -1.1)	<.001	-	-	-3.9 (-5.0, -2.9)	<.001	-3.2 (-3.6, -2.7)	<.001
		Male		-	-	-1.1 (-1.1, -1)	<.001	-2.1 (-2.4, -1.9)	<.001	-1.6 (-2.1, -1.1)	<.001	-	-	-2.2 (-2.7, -1.6)	<.001	-1.7 (-2.0, -1.4)	<.001
		Both	2010-2019	-	-	-0.4 (-0.7, -0.2)	.001	-0.1 (-0.3, 0.1)	.216	-0.4 (-0.7, -0.1)	.018	-	-	-2.7 (-3.2, -2.2)	<.001	-1.8 (-2.2, -1.4)	<.001
		Female		-	-	-1.1 (-1.4, -0.8)	<.001	0.0 (-0.3, 0.3)	.991	-0.2 (-0.6, 0.2)	.332	-	-	-2.9 (-3.6, -2.2)	<.001	-1.4 (-1.9, -1.0)	<.001
		Male		-	-	0.1 (-0.2, 0.3)	.475	-0.3 (-0.5, 0.0)	.018	-0.6 (-0.9, -0.4)	<.001	-	-	-2.6 (-3.2, -2.1)	<.001	-2.4 (-2.8, -2.0)	<.001
	African region	Both	1990-1999	-	-	-0.3 (-0.7, 0.0)	.027	0.2 (-0.2, 0.6)	.322	1.0 (0.8, 1.1)	<.001	-	-	-2.5 (-3.0, -2.0)	<.001	-0.5 (-1.8, 0.9)	.470
		Female		-	-	-1.4 (-1.7, -1.1)	<.001	0.1 (-0.7, 0.9)	.846	1.0 (0.7, 1.2)	<.001	-	-	-3.3 (-4.0, -2.6)	<.001	-1.6 (-3.9, 0.7)	.169
		Male		-	-	0.4 (0.2, 0.6)	.001	0.6 (0.2, 1.0)	.003	1.1 (0.9, 1.3)	<.001	-	-	-1.5 (-1.7, -1.3)	<.001	1.8 (1.4, 2.2)	<.001
		Both	2000-2009	-	-	-0.9 (-1, -0.7)	<.001	-1.1 (-1.2, -1.0)	<.001	1.4 (1, 1.7)	<.001	-	-	-3.8 (-3.9, -3.7)	<.001	-6.0 (-6.4, -5.7)	<.001
		Female		-	-	-1.9 (-2.1, -1.7)	<.001	-1.6 (-1.9, -1.3)	<.001	1.7 (1.4, 2.1)	<.001	-	-	-4.4 (-4.6, -4.2)	<.001	-5.2 (-5.8, -4.5)	<.001
		Male		-	-	-0.3 (-0.3, -0.2)	<.001	-0.1 (-0.1, 0.0)	.028	0.3 (0.3, 0.4)	<.001	-	-	-2.9 (-3.0, -2.8)	<.001	-7.5 (-8.1, -6.9)	<.001
		Both	2010-2019	-	-	-0.5 (-0.6, -0.4)	<.001	0.2 (0.0, 0.5)	.092	0.8 (0.6, 1.1)	<.001	-	-	-3.1 (-3.3, -2.9)	<.001	-1.8 (-2.8, -0.8)	.001
		Female		-	-	-1.5 (-1.6, -1.4)	<.001	0.1 (0.0, 0.3)	.091	1.0 (0.8, 1.3)	<.001	-	-	-3.5 (-3.7, -3.3)	<.001	-1.6 (-2.3, -0.9)	<.001
		Male		-	-	0.0 (-0.1, 0.0)	.203	0.5 (0.3, 0.7)	<.001	0.2 (0.2, 0.2)	<.001	-	-	-2.8 (-3.2, -2.4)	<.001	-3 (-3.7, -2.4)	<.001
	Eastern Mediterranean region	Both	1990-1999	-	-	1.0 (0.6, 1.4)	<.001	0.5 (0.3, 0.6)	<.001	1.0 (0.6, 1.5)	<.001	-	-	0.4 (0.0, 0.9)	.072	2.1 (1.9, 2.3)	<.001
		Female		-	-	0.9 (0.7, 1.1)	<.001	0.9 (0.7, 1.1)	<.001	1.1 (0.7, 1.5)	<.001	-	-	-0.3 (-0.8, 0.2)	.216	2.1 (1.9, 2.2)	<.001
		Male		-	-	1.1 (0.9, 1.4)	<.001	-0.3 (-0.5, -0.1)	.001	0.8 (0.3, 1.3)	.003	-	-	1.1 (0.7, 1.4)	<.001	2.2 (2.0, 2.3)	<.001
		Both	2000-2009	-	-	0 (-0.1, 0.1)	.935	-0.4 (-0.6, -0.2)	<.001	0.7 (0.4, 1.0)	<.001	-	-	-2.6 (-2.7, -2.5)	<.001	-0.1 (-0.3, 0.0)	.057
		Female		-	-	-0.4 (-0.5, -0.3)	<.001	-0.2 (-0.4, 0.1)	.218	0.7 (0.6, 0.9)	<.001	-	-	-3.0 (-3.2, -2.9)	<.001	-0.2 (-0.8, 0.4)	.528
		Male		-	-	0.5 (0.4, 0.6)	<.001	-0.8 (-0.9, -0.7)	<.001	0.6 (0.5, 0.8)	<.001	-	-	-2.3 (-2.4, -2.2)	<.001	0.0 (-0.2, 0.1)	.872
		Both	2010-2019	-	-	0.5 (0.1, 0.9)	.010	0.5 (0.2, 0.7)	<.001	-1.0 (-1.2, -0.8)	<.001	-	-	-0.8 (-1.1, -0.4)	<.001	0.1 (-0.2, 0.4)	.399
		Female		-	-	0.0 (-0.7, 0.6)	.887	0.6 (0.3, 0.9)	<.001	-1.0 (-1.2, -0.9)	<.001	-	-	-1.4 (-1.7, -1.0)	<.001	-0.3 (-0.6, 0)	.045
		Male		-	-	1.0 (0.7, 1.3)	<.001	0.2 (0.1, 0.3)	.001	-1.0 (-1.2, -0.7)	<.001	-	-	-0.4 (-0.8, -0.1)	.018	0.8 (0.6, 1.1)	<.001
	European region	Both	1990-1999	-	-	3.0 (2.2, 3.8)	<.001	-0.2 (-0.8, 0.4)	.549	-0.3 (-0.7, 0.1)	.100	-	-	-0.7 (-1.5, 0.1)	.104	0.1 (-0.4, 0.6)	.811
		Female		-	-	2.6 (2.0, 3.2)	<.001	0.1 (-0.4, 0.7)	.609	-0.5 (-0.8, -0.3)	<.001	-	-	-1.1 (-2.1, 0.0)	.042	0.2 (-0.2, 0.7)	.328
		Male		-	-	3.1 (2.1, 4.0)	<.001	-0.3 (-0.6, 0)	.061	-0.1 (-0.7, 0.5)	.715	-	-	-0.1 (-0.9, 0.6)	.704	-0.2 (-0.5, 0.1)	.244
		Both	2000-2009	-	-	-3.1 (-3.8, -2.5)	<.001	-1.1 (-1.3, -1)	<.001	-2.2 (-2.5, -1.8)	<.001	-	-	-5.6 (-7.2, -4.0)	<.001	-3.2 (-3.6, -2.9)	<.001
		Female		-	-	-3.1 (-3.6, -2.6)	<.001	-0.7 (-0.8, -0.6)	<.001	-1.8 (-1.9, -1.6)	<.001	-	-	-5.1 (-6.0, -4.2)	<.001	-2.8 (-3.1, -2.6)	<.001
		Male		-	-	-2.9 (-3.7, -2.2)	<.001	-1.7 (-3.1, -0.3)	.018	-2.7 (-3.2, -2.2)	<.001	-	-	-6.2 (-8.1, -4.2)	<.001	-4.0 (-4.7, -3.3)	<.001
		Both	2010-2019	-	-	-0.3 (-0.8, 0.3)	.374	0 (-0.4, 0.4)	.843	-0.6 (-1.1, -0.1)	.025	-	-	-3.6 (-4.4, -2.9)	<.001	-0.8 (-1.1, -0.5)	<.001
		Female		-	-	-0.9 (-1.5, -0.4)	.002	-0.3 (-0.9, 0.3)	.360	-0.7 (-1.0, -0.4)	<.001	-	-	-3.5 (-4.4, -2.6)	<.001	-0.8 (-1.1, -0.5)	<.001
		Male		-	-	0.3 (-0.4, 1.0)	.375	0.1 (-0.2, 0.5)	.452	-0.4 (-1.2, 0.4)	.339	-	-	-3.6 (-4.2, -3.0)	<.001	-0.8 (-1.2, -0.4)	<.001
	Region of the Americas	Both	1990-1999	-	-	0.5 (0, 1.0)	.041	-1.1 (-1.2, -1.0)	<.001	0.8 (0.6, 1.0)	<.001	-	-	-4.8 (-5.2, -4.4)	<.001	-1.6 (-1.7, -1.5)	<.001
		Female		-	-	0.5 (0.0, 1.1)	.054	-1.3 (-1.5, -1.1)	<.001	0.9 (0.6, 1.2)	<.001	-	-	-4.9 (-5.2, -4.7)	<.001	-1.2 (-1.4, -1.0)	<.001
		Male		-	-	0.5 (0.0, 1.0)	.067	-0.7 (-0.8, -0.5)	<.001	0.5 (0.0, 1.1)	.057	-	-	-4.6 (-5.1, -4.2)	<.001	-2.2 (-2.7, -1.8)	<.001
		Both	2000-2009	-	-	0.2 (0.1, 0.2)	<.001	1.4 (1.3, 1.5)	<.001	0.5 (0.4, 0.7)	<.001	-	-	-4.3 (-4.5, -4.1)	<.001	0.2 (-0.2, 0.6)	.259
		Female		-	-	0.3 (0.2, 0.4)	<.001	1.5 (0.8, 2.2)	<.001	0.4 (0.1, 0.7)	.003	-	-	-4.5 (-4.7, -4.3)	<.001	0.5 (-0.2, 1.1)	.167
		Male		-	-	0.0 (-0.1, 0.2)	.819	1.3 (1.1, 1.6)	<.001	0.9 (0.7, 1.1)	<.001	-	-	-4.3 (-4.5, -4.0)	<.001	-0.5 (-0.7, -0.3)	<.001
		Both	2010-2019	-	-	0.1 (-0.4, 0.5)	.671	0.4 (0.0, 0.9)	.071	-0.4 (-0.8, 0.0)	.031	-	-	-2.5 (-2.9, -2.2)	<.001	-0.2 (-0.7, 0.2)	.275
		Female		-	-	0.0 (-0.7, 0.7)	.988	0.9 (0.0, 1.8)	.044	-0.5 (-0.7, -0.2)	<.001	-	-	-2.5 (-2.9, -2.1)	<.001	0.0 (-0.9, 0.8)	.932
		Male		-	-	0.2 (-0.3, 0.7)	.377	0.1 (-0.4, 0.6)	.644	-0.4 (-1.0, 0.3)	.301	-	-	-2.6 (-2.9, -2.3)	<.001	-0.7 (-1.1, -0.3)	<.001
	South-East Asia region	Both	1990-1999	-	-	-1.8 (-2.4, -1.1)	<.001	-0.4 (-0.6, -0.2)	<.001	0.9 (0.6, 1.2)	<.001	-	-	-0.3 (-1.7, 1.0)	.634	0.7 (0.5, 0.9)	<.001
		Female		-	-	-2.5 (-3, -1.9)	<.001	-0.5 (-0.9, -0.2)	.005	0.5 (0.2, 0.8)	.001	-	-	-0.8 (-1.9, 0.3)	.172	0.5 (-0.1, 1.2)	.079
		Male		-	-	-0.9 (-1.6, -0.2)	.011	-0.2 (-0.4, 0.0)	.023	1.9 (1.5, 2.3)	<.001	-	-	-0.1 (-1.4, 1.2)	.894	0.9 (0.3, 1.5)	.003
		Both	2000-2009	-	-	-1.8 (-2.4, -1.1)	<.001	-3.6 (-4.4, -2.8)	<.001	-1.5 (-2.3, -0.6)	.001	-	-	-3.0 (-3.6, -2.3)	<.001	-1.9 (-2.5, -1.4)	<.001
		Female		-	-	-2.5 (-3.0, -1.9)	<.001	-3.6 (-4.2, -3.0)	<.001	-1.6 (-2.4, -0.7)	<.001	-	-	-3.9 (-5.7, -2.0)	<.001	-2.0 (-2.4, -1.5)	<.001
		Male		-	-	-0.9 (-1.6, -0.2)	.011	-3.2 (-3.5, -2.8)	<.001	-1.0 (-1.5, -0.4)	.001	-	-	-2.5 (-3.4, -1.6)	<.001	-1.8 (-2.7, -0.9)	<.001
		Both	2010-2019	-	-	-1.8 (-2.4, -1.1)	<.001	-1.7 (-2.3, -1.1)	<.001	-0.4 (-1.0, 0.2)	.157	-	-	-4.3 (-5.2, -3.4)	<.001	-1.4 (-1.9, -0.9)	<.001
		Female		-	-	-2.5 (-3.0, -1.9)	<.001	-1.7 (-2.8, -0.6)	.002	-0.2 (-0.7, 0.4)	.536	-	-	-4.1 (-5.3, -2.9)	<.001	-0.9 (-1.6, -0.2)	.007
		Male		-	-	-0.9 (-1.6, -0.2)	.011	-1.8 (-2.2, -1.4)	<.001	-0.6 (-1.3, 0.1)	.078	-	-	-4.5 (-5.4, -3.6)	<.001	-2.3 (-2.9, -1.7)	<.001
	Western Pacific region	Both	1990-1999	-	-	1.0 (-0.3, 2.2)	.119	-1.3 (-1.7, -1.0)	<.001	2.7 (0.5, 5.1)	.019	-	-	-4.0 (-4.4, -3.6)	<.001	-1.7 (-3.3, -0.2)	.031
		Female		-	-	3.0 (1.9, 4.2)	<.001	0.8 (-0.2, 1.9)	.097	4.4 (2.1, 6.7)	<.001	-	-	-3.1 (-3.8, -2.3)	<.001	-0.8 (-1.4, -0.1)	.020
		Male		-	-	0.2 (-0.1, 0.5)	.230	-2.7 (-2.8, -2.6)	<.001	-0.3 (-1.5, 0.8)	.567	-	-	-6.0 (-6.8, -5.2)	<.001	-3.0 (-3.8, -2.1)	<.001
		Both	2000-2009	-	-	-5.3 (-5.8, -4.8)	<.001	-6.8 (-7.2, -6.4)	<.001	-5.7 (-7.6, -3.8)	<.001	-	-	-5.2 (-6.1, -4.4)	<.001	-2.4 (-2.8, -2.1)	<.001
		Female		-	-	-5.8 (-6.8, -4.8)	<.001	-8.2 (-9.0, -7.3)	<.001	-6.7 (-8.6, -4.8)	<.001	-	-	-7.4 (-8.8, -6.1)	<.001	-4.7 (-6.4, -2.8)	<.001
		Male		-	-	-4.1 (-4.6, -3.6)	<.001	-5.1 (-5.6, -4.5)	<.001	-3.4 (-3.7, -3.1)	<.001	-	-	-2.4 (-2.7, -2.2)	<.001	1.4 (0.1, 2.7)	.035
		Both	2010-2019	-	-	-0.5 (-1.0, 0.1)	.122	-1.8 (-2.7, -1.0)	<.001	-0.1 (-0.4, 0.2)	.357	-	-	-1.9 (-2.5, -1.2)	<.001	-2.4 (-3.1, -1.6)	<.001
		Female		-	-	-1.5 (-3.0, 0.0)	.057	-3.2 (-4.1, -2.3)	<.001	-0.1 (-0.4, 0.2)	.544	-	-	-2.5 (-3.6, -1.4)	<.001	-2.3 (-3.3, -1.3)	<.001
		Male		-	-	0.4 (0.2, 0.7)	<.001	-1.2 (-1.7, -0.7)	<.001	0.6 (0.1, 1.1)	.025	-	-	-1.1 (-1.4, -0.8)	<.001	-2.7 (-4.2, -1.3)	<.001
ASDR	Global	Both	1990-1999	-0.2 (-0.2, -0.2)	<.001	1.1 (1, 1.3)	<.001	0.1 (0.1, 0.2)	<.001	0.2 (0.0, 0.4)	.014	-1 (-1, -0.9)	<.001	-0.5 (-0.9, 0.0)	.038	0.1 (0.0, 0.1)	.125
		Female		-0.1 (-0.2, -0.1)	<.001	1.1 (0.9, 1.3)	<.001	0.2 (0.1, 0.3)	.001	0.4 (0.2, 0.6)	<.001	-1 (-1, -1)	<.001	-0.8 (-1.2, -0.4)	<.001	0.1 (0.0, 0.2)	.217
		Male		-0.3 (-0.3, -0.3)	<.001	1.2 (1.0, 1.3)	<.001	0.1 (0.0, 0.2)	.003	-0.2 (-0.4, 0.0)	.092	-0.9 (-0.9, -0.9)	<.001	-0.3 (-0.9, 0.3)	.349	0.0 (-0.1, 0.1)	.776
		Both	2000-2009	-0.1 (-0.1, -0.1)	<.001	-1.0 (-1.0, -0.9)	<.001	-1.4 (-1.5, -1.2)	<.001	-0.7 (-0.8, -0.5)	<.001	-1 (-1, -0.9)	<.001	-2.0 (-2.3, -1.8)	<.001	0.1 (0.1, 0.2)	<.001
		Female		-0.1 (-0.1, 0.0)	<.001	-1.7 (-1.9, -1.5)	<.001	-1.5 (-1.7, -1.2)	<.001	-0.7 (-0.8, -0.5)	<.001	-1 (-1, -1)	<.001	-2.7 (-3.4, -2.1)	<.001	0.1 (0.0, 0.1)	.001
		Male		-0.2 (-0.2, -0.1)	<.001	-0.6 (-0.6, -0.5)	<.001	-1.3 (-1.6, -1.1)	<.001	-0.8 (-1.0, -0.5)	<.001	-0.9 (-0.9, -0.9)	<.001	-1.4 (-1.8, -1.0)	<.001	0.2 (0.2, 0.3)	<.001
		Both	2010-2019	-0.1 (-0.1, -0.1)	<.001	0.0 (-0.2, 0.2)	.758	-0.5 (-0.6, -0.4)	<.001	-0.3 (-0.5, -0.2)	<.001	-0.9 (-0.9, -0.8)	<.001	-1.6 (-2.0, -1.1)	<.001	-0.1 (-0.4, 0.1)	.326
		Female		-0.1 (-0.1, -0.1)	<.001	-0.4 (-0.7, -0.1)	.009	-0.5 (-0.6, -0.3)	<.001	-0.2 (-0.3, -0.1)	.005	-1 (-1, -0.9)	<.001	-1.6 (-2.2, -0.9)	<.001	-0.1 (-0.4, 0.1)	.320
		Male		-0.1 (-0.1, -0.1)	<.001	0.4 (0.2, 0.6)	.001	-0.6 (-0.7, -0.5)	<.001	-0.4 (-0.5, -0.3)	<.001	-0.8 (-0.8, -0.7)	<.001	-1.8 (-2.2, -1.3)	<.001	0.0 (-0.3, 0.3)	.812
	African region	Both	1990-1999	0.0 (0.0, 0.0)	<.001	-0.2 (-0.5, 0.0)	.044	0.2 (-0.2, 0.6)	.260	0.6 (0.5, 0.7)	<.001	-0.6 (-0.7, -0.6)	<.001	-1.3 (-1.6, -1.0)	<.001	0.0 (-0.5, 0.4)	.855
		Female		0.0 (0.0, 0.0)	.300	-1 (-1.2, -0.8)	<.001	0.0 (-0.6, 0.7)	.900	0.6 (0.5, 0.7)	<.001	-0.7 (-0.8, -0.6)	<.001	-1.8 (-2.1, -1.5)	<.001	-0.5 (-0.7, -0.4)	<.001
		Male		0.0 (0.0, 0.0)	.472	0.4 (0.2, 0.5)	<.001	0.6 (0.2, 1.0)	.003	0.6 (0.6, 0.7)	<.001	-0.6 (-0.6, -0.5)	<.001	-0.7 (-0.9, -0.6)	<.001	0.7 (0.5, 0.9)	<.001
		Both	2000-2009	0.0 (0.0, 0.0)	<.001	-0.6 (-0.8, -0.5)	<.001	-1.0 (-1.0, -0.9)	<.001	0.9 (0.8, 0.9)	<.001	-0.5 (-0.5, -0.5)	<.001	-1.7 (-1.8, -1.7)	<.001	-0.7 (-0.9, -0.6)	<.001
		Female		0.0 (0.0, 0.0)	<.001	-1.3 (-1.4, -1.1)	<.001	-1.5 (-1.6, -1.3)	<.001	1.2 (1.1, 1.3)	<.001	-0.5 (-0.5, -0.5)	<.001	-2.1 (-2.2, -1.9)	<.001	-0.5 (-0.7, -0.4)	<.001
		Male		0.0 (0.0, 0.0)	<0.001	-0.2 (-0.3, -0.1)	<.001	-0.1 (-0.1, 0.0)	.015	0.2 (0.2, 0.2)	<.001	-0.5 (-0.5, -0.4)	<.001	-1.3 (-1.3, -1.2)	<.001	-1.6 (-1.9, -1.3)	<.001
		Both	2010-2019	0.0 (0.0, 0.0)	<0.001	-0.3 (-0.4, -0.2)	<.001	0.2 (0.0, 0.4)	.092	0.4 (0.3, 0.4)	<.001	-0.9 (-1, -0.9)	<.001	-1.1 (-1.2, -1.0)	<.001	-0.8 (-1.1, -0.6)	<.001
		Female		0.0 (0.0, 0.0)	<0.001	-0.9 (-1.0, -0.8)	<.001	0.1 (-0.1, 0.2)	.355	0.5 (0.5, 0.5)	<.001	-0.9 (-1, -0.8)	<.001	-1.2 (-1.3, -1.1)	<.001	-0.5 (-0.7, -0.4)	<.001
		Male		0.0 (0.0, 0.0)	<0.001	0.0 (0.0, 0.1)	.090	0.4 (0.2, 0.6)	<.001	-0.1 (-0.1, 0.0)	<.001	-0.9 (-1.1, -0.8)	<.001	-1.0 (-1.3, -0.7)	<.001	-0.3 (-0.4, -0.1)	<.001
	Eastern Mediterranean region	Both	1990-1999	0.1 (0.0, 0.1)	<0.001	1.1 (0.8, 1.4)	<.001	0.5 (0.4, 0.6)	<.001	0.4 (0.3, 0.5)	<.001	-0.9 (-0.9, -0.8)	<.001	0.4 (0.2, 0.6)	<.001	0.2 (0.1, 0.3)	.003
		Female		0.0 (0.0, 0.0)	<0.001	0.9 (0.7, 1.2)	<.001	0.7 (0.6, 0.9)	<.001	0.5 (0.3, 0.6)	<.001	-0.9 (-0.9, -0.8)	<.001	-0.1 (-0.4, 0.2)	.447	0.3 (0.2, 0.5)	<.001
		Male		0.0 (0.0, 0.0)	<0.001	1.2 (1.0, 1.3)	<.001	0.2 (0.1, 0.3)	<.001	0.2 (0.0, 0.3)	.051	-0.9 (-0.9, -0.8)	<.001	1.0 (0.7, 1.4)	<.001	-0.1 (-0.2, 0.0)	.056
		Both	2000-2009	0.0 (0.0, 0.0)	0.002	0.6 (0.5, 0.7)	<.001	-0.2 (-0.3, -0.1)	<.001	0.5 (0.5, 0.6)	<.001	-1.1 (-1.1, -1)	<.001	-2.2 (-2.3, -2.1)	<.001	0.4 (0.4, 0.5)	<.001
		Female		0.0 (0.0, 0.0)	<0.001	0.2 (-0.1, 0.5)	.122	-0.1 (-0.2, 0.1)	.341	0.5 (0.5, 0.6)	<.001	-1 (-1.1, -1)	<.001	-2.5 (-2.6, -2.4)	<.001	0.5 (0.4, 0.5)	<.001
		Male		0.0 (0.0, 0.0)	<0.001	0.8 (0.5, 1.0)	<.001	-0.4 (-0.5, -0.4)	<.001	0.5 (0.5, 0.6)	<.001	-1.1 (-1.2, -1.1)	<.001	-1.9 (-2, -1.8)	<.001	0.4 (0.3, 0.5)	<.001
		Both	2010-2019	0.0 (0.0, 0.0)	0.037	1.0 (0.8, 1.1)	<.001	0.2 (0.0, 0.3)	.009	-0.5 (-0.7, -0.4)	<.001	-0.8 (-0.8, -0.8)	<.001	-0.5 (-0.8, -0.2)	.001	0.9 (0.9, 0.9)	<.001
		Female		0.0 (0.0, 0.0)	<0.001	0.6 (0.4, 0.7)	<.001	0.3 (0.1, 0.5)	.001	-0.6 (-0.7, -0.6)	<.001	-0.8 (-0.9, -0.8)	<.001	-0.9 (-1.4, -0.5)	<.001	0.6 (0.5, 0.7)	<.001
		Male		0.0 (0.0, 0.0)	<0.001	1.4 (1.3, 1.4)	<.001	0.0 (-0.3, 0.2)	.778	-0.5 (-0.6, -0.5)	<.001	-0.7 (-0.8, -0.7)	<.001	-0.3 (-0.7, 0.0)	.080	1.3 (1.2, 1.4)	<.001
	European region	Both	1990-1999	-0.1 (-0.1, -0.1)	<0.001	2.6 (2.0, 3.1)	<.001	1.0 (0.8, 1.1)	<.001	0.1 (-0.1, 0.4)	.290	-0.9 (-0.9, -0.9)	<.001	-0.6 (-1.2, 0.1)	.105	0.3 (0.1, 0.4)	<.001
		Female		0.0 (0.0, 0.0)	0.251	2.5 (2.0, 2.9)	<.001	1.0 (0.7, 1.3)	<.001	0.1 (-0.1, 0.3)	.312	-0.9 (-0.9, -0.8)	<.001	-0.8 (-1.7, 0.1)	.065	0.2 (0.2, 0.3)	<.001
		Male		-0.3 (-0.3, -0.2)	<0.001	2.6 (2.0, 3.2)	<.001	0.9 (0.8, 1.0)	<.001	0.1 (-0.2, 0.5)	.462	-0.9 (-1, -0.9)	<.001	-0.1 (-0.7, 0.5)	.755	0.2 (0.1, 0.4)	.003
		Both	2000-2009	0.0 (-0.1, 0.0)	0.022	-1.3 (-1.5, -1.1)	<.001	-0.4 (-0.5, -0.3)	<.001	-0.9 (-1.0, -0.8)	<.001	-0.5 (-0.6, -0.5)	<.001	-4.5 (-5.8, -3.1)	<.001	0.1 (0.1, 0.2)	<.001
		Female		0.0 (0.0, 0.0)	0.209	-1.5 (-1.7, -1.3)	<.001	-0.3 (-0.4, -0.3)	<.001	-0.6 (-0.6, -0.5)	<.001	-0.6 (-0.6, -0.5)	<.001	-3.9 (-4.4, -3.5)	<.001	0.2 (0.2, 0.3)	<.001
		Male		-0.1 (-0.1, 0.0)	<0.001	-1.1 (-1.3, -0.9)	<.001	-0.5 (-0.8, -0.2)	.001	-1.2 (-1.5, -0.9)	<.001	-0.6 (-0.6, -0.5)	<.001	-4.9 (-6.4, -3.5)	<.001	-0.1 (-0.2, -0.1)	<.001
		Both	2010-2019	0.1 (0.0, 0.1)	<0.001	0.3 (-0.1, 0.8)	.179	0.3 (0.2, 0.4)	<.001	0.1 (-0.1, 0.2)	.503	0.1 (0, 0.1)	.009	-2.4 (-3.0, -1.8)	<.001	0.5 (0.4, 0.6)	<.001
		Female		-0.1 (-0.1, 0.0)	<0.001	0.0 (-0.4, 0.4)	.944	0.2 (0.1, 0.3)	<.001	0.2 (0.1, 0.3)	.001	0 (0, 0.1)	.579	-2.3 (-3.2, -1.5)	<.001	0.6 (0.5, 0.7)	<.001
		Male		0.3 (0.2, 0.3)	<0.001	0.6 (0.1, 1.1)	.016	0.4 (0.3, 0.5)	<.001	0 (-0.4, 0.4)	.866	0.1 (0.1, 0.2)	<.001	-2.5 (-3.1, -1.9)	<.001	0.5 (0.3, 0.6)	<.001
	Region of the Americas	Both	1990-1999	-0.5 (-0.6, -0.5)	<0.001	0.2 (0.0, 0.3)	.018	-2.2 (-2.3, -2.1)	<.001	-0.1 (-0.2, 0.0)	.052	-0.2 (-0.2, -0.1)	<.001	-2.5 (-2.7, -2.3)	<.001	0.5 (0.3, 0.6)	<.001
		Female		-0.3 (-0.4, -0.3)	<0.001	0.3 (0.0, 0.6)	.087	-2.3 (-2.5, -2.2)	<.001	-0.1 (-0.2, 0.0)	.006	-0.2 (-0.3, -0.1)	<.001	-2.6 (-2.8, -2.4)	<.001	0.5 (0.4, 0.6)	<.001
		Male		-0.9 (-0.9, -0.8)	<0.001	0.3 (0.2, 0.3)	<.001	-1.9 (-2, -1.7)	<.001	-0.1 (-0.2, 0.1)	.316	-0.1 (-0.2, -0.1)	<.001	-2.3 (-2.5, -2.1)	<.001	0.4 (0.4, 0.5)	<.001
		Both	2000-2009	0.0 (0.0, 0.0)	0.050	0.3 (0.2, 0.5)	<.001	0.5 (0.3, 0.6)	<.001	0.2 (0.1, 0.3)	.005	-1.3 (-1.3, -1.2)	<.001	-1.8 (-1.9, -1.7)	<.001	0.6 (0.5, 0.7)	<.001
		Female		0.0 (0.0, 0.0)	0.869	0.5 (0.4, 0.6)	<.001	0.6 (0.4, 0.8)	<.001	0.2 (0.2, 0.3)	<.001	-1.3 (-1.3, -1.3)	<.001	-1.8 (-2, -1.6)	<.001	0.7 (0.6, 0.7)	<.001
		Male		0 (-0.1, 0.0)	0.006	0.2 (0.1, 0.2)	<.001	0.4 (0.4, 0.5)	<.001	0.2 (0.0, 0.4)	.074	-1.2 (-1.3, -1.1)	<.001	-1.8 (-1.9, -1.7)	<.001	0.6 (0.5, 0.7)	<.001
		Both	2010-2019	0.0 (0.0, 0.0)	<0.001	0.2 (-0.1, 0.5)	.133	-0.2 (-0.3, -0.1)	.006	-0.1 (-0.1, 0.0)	.001	-0.4 (-0.5, -0.4)	<.001	-0.9 (-1, -0.8)	<.001	0.3 (0.3, 0.4)	<.001
		Female		0.0 (0.0, 0.0)	0.261	0.2 (-0.3, 0.7)	.439	0.0 (-0.2, 0.2)	.853	-0.1 (-0.1, 0.0)	.029	-0.4 (-0.5, -0.3)	<.001	-0.9 (-1, -0.7)	<.001	0.4 (0.3, 0.4)	<.001
		Male		0.0 (0.0, 0.0)	0.013	0.3 (-0.3, 0.8)	.373	-0.3 (-0.4, -0.3)	<.001	-0.1 (-0.2, 0.1)	.251	-0.5 (-0.5, -0.4)	<.001	-0.8 (-1, -0.7)	<.001	0.2 (0.1, 0.3)	<.001
	South-East Asia region	Both	1990-1999	0.0 (0.0, 0.0)	<0.001	0.5 (-0.6, 1.5)	.382	-0.3 (-0.5, -0.1)	.003	0.7 (0.5, 0.9)	<.001	0.3 (0.3, 0.3)	<.001	-0.4 (-1.5, 0.7)	.473	0.5 (0.4, 0.5)	<.001
		Female		0.0 (-0.1, 0.0)	<0.001	-0.1 (-0.8, 0.5)	.645	-0.4 (-0.7, -0.1)	.013	0.5 (0.3, 0.6)	<.001	0.3 (0.2, 0.4)	<.001	-0.8 (-1.7, 0.1)	.088	0.5 (0.4, 0.5)	<.001
		Male		-0.1 (-0.1, 0.0)	<0.001	1.0 (-0.1, 2.2)	.088	-0.1 (-0.3, 0.1)	.188	1.3 (1.0, 1.5)	<.001	0.3 (0.3, 0.3)	<.001	-0.2 (-1.5, 1.1)	.766	0.4 (0.4, 0.4)	<.001
		Both	2000-2009	-0.1 (-0.1, -0.1)	<0.001	-1.4 (-1.6, -1.2)	<.001	-2.9 (-3.6, -2.1)	<.001	-0.8 (-1.3, -0.3)	.001	0 (0, 0.1)	<.001	-2.4 (-3, -1.9)	<.001	0.4 (0.4, 0.5)	<.001
		Female		-0.1 (-0.1, -0.1)	<0.001	-2.5 (-2.9, -2.1)	<.001	-2.9 (-3.5, -2.4)	<.001	-0.9 (-1.4, -0.3)	.001	0 (0, 0.1)	.126	-3.1 (-4.6, -1.6)	<.001	0.5 (0.4, 0.5)	<.001
		Male		-0.1 (-0.1, -0.1)	<0.001	-0.6 (-0.8, -0.3)	<.001	-2.5 (-2.8, -2.2)	<.001	-0.5 (-0.8, -0.1)	.008	0.1 (0.1, 0.1)	<.001	-2.1 (-2.9, -1.2)	<.001	0.5 (0.5, 0.5)	<.001
		Both	2010-2019	-0.1 (-0.1, -0.1)	<0.001	-1.4 (-1.9, -0.8)	<.001	-1.6 (-2.1, -1.1)	<.001	-0.1 (-0.5, 0.2)	.422	-0.4 (-0.5, -0.3)	<.001	-3.5 (-4.3, -2.6)	<.001	0.6 (0.6, 0.7)	<.001
		Female		-0.1 (-0.1, -0.1)	<0.001	-1.7 (-2.4, -1.1)	<.001	-1.5 (-2.3, -0.7)	<.001	0.0 (-0.4, 0.3)	.911	-0.4 (-0.5, -0.3)	<.001	-3.4 (-4.5, -2.2)	<.001	0.7 (0.6, 0.7)	<.001
		Male		-0.1 (-0.1, -0.1)	<0.001	-1.2 (-2.0, -0.4)	.003	-1.8 (-2.2, -1.4)	<.001	-0.2 (-0.7, 0.2)	.309	-0.4 (-0.5, -0.3)	<.001	-3.7 (-4.6, -2.8)	<.001	0.6 (0.6, 0.6)	<.001
	Western Pacific region	Both	1990-1999	0.1 (0.0, 0.1)	0.003	0.9 (-0.2, 2.0)	.116	2.9 (2.8, 3.1)	<.001	2.1 (1.0, 3.2)	<.001	-1.2 (-1.2, -1.1)	<.001	-4.0 (-4.1, -3.8)	<.001	0.0 (-0.2, 0.2)	.967
		Female		0.0 (0.0, 0.0)	<0.001	1.9 (0.1, 3.8)	.038	4.1 (3.8, 4.5)	<.001	3.3 (1.9, 4.8)	<.001	-1.1 (-1.1, -1)	<.001	-3.1 (-3.6, -2.6)	<.001	0.1 (0.0, 0.1)	.005
		Male		0.1 (0.1, 0.2)	<0.001	0.3 (0.0, 0.6)	.092	2.4 (2.1, 2.8)	<.001	-0.2 (-0.9, 0.6)	.616	-1.3 (-1.3, -1.2)	<.001	-5.2 (-5.3, -5)	<0.001	-0.2 (-0.3, 0.0)	0.044
		Both	2000-2009	0.0 (0.0, 0.0)	0.001	-3.9 (-4.2, -3.5)	<.001	-2.6 (-2.8, -2.4)	<.001	-4.0 (-4.9, -3.0)	<.001	-0.9 (-1, -0.8)	<.001	-3.1 (-3.2, -2.9)	<.001	0.4 (0.4, 0.5)	<.001
		Female		0.0 (0.0, 0.0)	<0.001	-4.5 (-5.2, -3.9)	<.001	-2.9 (-3.2, -2.5)	<.001	-4.8 (-6.0, -3.6)	<.001	-0.9 (-0.9, -0.8)	<.001	-4.8 (-5.9, -3.6)	<.001	0.2 (0.1, 0.2)	<.001
		Male		0.0 (-0.1, 0.0)	0.394	-3.0 (-3.6, -2.5)	<.001	-1.9 (-2.1, -1.6)	<.001	-2.2 (-2.4, -2.0)	<.001	-0.9 (-1, -0.8)	<.001	-1.3 (-1.5, -1.1)	<.001	0.8 (0.7, 0.9)	<.001
		Both	2010-2019	0.0 (0.0, 0.0)	0.142	0.2 (-0.3, 0.7)	.426	-0.4 (-0.6, -0.1)	.002	0.3 (0.1, 0.5)	.002	-1 (-1.1, -1)	<.001	-1.3 (-1.6, -1)	<.001	-1.0 (-1.5, -0.4)	.001
		Female		0.0 (0.0, 0.0)	0.188	-0.5 (-1.6, 0.5)	.328	-0.6 (-1.2, -0.1)	.031	0.2 (0.1, 0.4)	.013	-1 (-1, -0.9)	<.001	-1.7 (-2, -1.4)	<.001	-0.9 (-1.6, -0.2)	.012
		Male		0.1 (0.1, 0.2)	<0.001	0.7 (0.5, 0.9)	<.001	-0.2 (-0.3, -0.1)	.004	0.7 (0.4, 0.9)	<.001	-1 (-1.1, -0.9)	<.001	-0.6 (-0.9, -0.4)	<.001	-0.8 (-1.4, -0.2)	.011

AA, alopecia areata; AAPC, average annual percentage change; ADs, autoimmune diseases; ASDR, Age-standardized Disability-adjusted Life Year; ASIR, age-standardized incidence rate; ASMR, age-standardized mortality rate; ASPR, age-standardized prevalence rate; IBD, inflammatory bowel disease; MS, multiple sclerosis; RA, rheumatoid arthritis; RHD, rheumatic heart disease; T1DM, type 1 diabetes mellitus.

## Data Availability

Data utilized in this study can be obtained from the Global Burden of Disease Study 2019 (GBD 2019) (https://ghdx.healthdata.org/gbd-2019).
