# Supplementary material for: Temporal Trend and Health Inequality in the Burden of Autoimmune Diseases Among Older Adolescents and Young Adults Aged 15-29 Years
Source: Arch Rheumatol. 2025 Sep 1;40(3):332–57. doi: 10.5152/ArchRheumatol.2025.11174 (PMC12502848; doi:10.5152/ArchRheumatol.2025.11174)
Supplement: Supplementary Material [file supplementary_material.pdf]

Supplementary Tables 1-7. https://docs.google.com/spreadsheets/d/1eoMX-YMo1ct_uVzmTcTMHQtm1d9a_cVFpZyB3IhC0qE/edit?gid=0#gid=0

Supplementary Figures 1-12. https://docs.google.com/spreadsheets/d/1TrYNzNZgRc9nE76dZ-s7nOF27syi6f1zWM2U5nPxWMM/edit?usp=sharing
